# GTP binding to translation factor eIF2B stimulates its guanine nucleotide exchange activity

**DOI:** 10.1016/j.isci.2021.103454

**Published:** 2021-11-14

**Authors:** Christopher J. Kershaw, Martin D. Jennings, Francesco Cortopassi, Margherita Guaita, Hawra Al-Ghafli, Graham D. Pavitt

**Affiliations:** 1 Division of Molecular and Cellular Function, School of Biological Sciences, Faculty of Biology, Medicine and Health, The University of Manchester, Manchester M13 9PT, UK

**Keywords:** Biological sciences, Molecular biology, Cell biology, Biomechanics

## Abstract

eIF2B is the guanine nucleotide exchange factor (GEF) required for cytoplasmic protein synthesis initiation in eukaryotes and its regulation within the integrated stress response (ISR). It activates its partner factor eIF2, thereby promoting translation initiation. Here we provide evidence through biochemical and genetic approaches that eIF2B can bind directly to GTP and this can enhance its rate of GEF activity toward eIF2–GDP *in vitro*. GTP binds to a subcomplex of the eIF2Bγ and ε subunits. The eIF2Bγ amino-terminal domain shares structural homology with hexose sugar phosphate pyrophosphorylase enzymes that bind specific nucleotides. A K66R mutation in eIF2Bγ is especially sensitive to guanine or GTP in a range of functional assays. Taken together, our data suggest eIF2Bγ may act as a sensor of purine nucleotide availability and thus modulate eIF2B activity and protein synthesis in response to fluctuations in cellular nucleotide levels.

## Highlights

• eIF2B, the GDP exchange factor for eIF2 in translation and its control, binds GTP
• GTP binding enhances the rate of eIF2B GEF activity toward eIF2–GDP *in vitro*
• A K66R mutation in yeast eIF2Bγ is sensitive to guanine *in vivo* or GTP *in vitro*
• eIF2B may act as a sensor of purine nucleotide availability

## Graphical abstract

**Figure:**
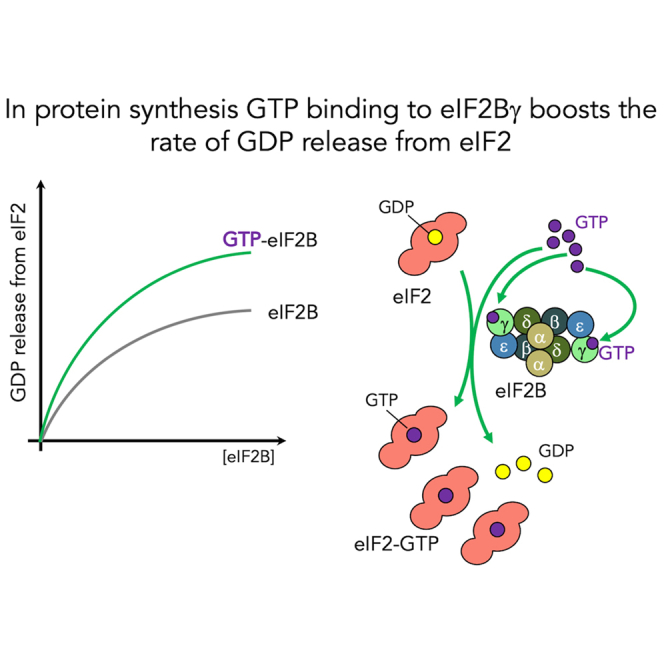

## Introduction

Translation initiation is a highly regulated phase of gene expression. Methionine initiator tRNA (Met-tRNAi) is brought to the small ribosomal subunit (40S) as part of a ternary complex (TC) with the GTP-binding translation initiation factor eIF2 in complex with GTP. Several other initiation factors assist as part of a preinitiation complex (PIC), which then binds to mRNA near its 5′ cap and scans to an AUG codon where codon-anticodon pairing signals selection of the start site. The action of the GTPase-activating protein eIF5 facilitates GTP hydrolysis to GDP and inorganic phosphate (Pi) within the TC. Pi release from eIF2 reduces by ∼1,000 fold its affinity for Met-tRNAi, enabling eIF2–GDP/eIF5 release from the PIC, which consequentially facilitates 60S joining for translation elongation to begin (Hinnebusch, 2014; Merrick and Pavitt, 2018).

For continued rounds of initiation, the relatively stable eIF2-GDP complex must be reactivated into eIF2-GTP for Met-tRNAi binding. This recycling of eIF2 is performed by the guanine nucleotide exchange factor (GEF) eIF2B. Because eIF2-GTP is unstable, Met-tRNAi and eIF5 binding is likely closely coupled to GEF activity (Jennings et al., 2017). Hence, the activation of eIF2 by eIF2B can be viewed as the first step of protein synthesis (Merrick and Pavitt, 2018). As with the first steps of many biochemical pathways, eIF2B activity is highly regulated. In particular its GEF activity is dramatically inhibited by phosphorylation of the α subunit of its substrate eIF2. In the yeast *Saccharomyces cerevisiae*, a sole eIF2α kinase Gcn2 is activated by stresses that promote ribosome stalling during elongation, such as amino acid limitation, leading to de-acylated tRNA accumulation (Hinnebusch, 2005; Pochopien et al., 2021). In mammals, additional kinases (i.e. PKR, PERK, and HRI) respond to a wider range of cellular stresses (Pavitt, 2018; Wek, 2018). This phosphorylation at a common serine (ser 52) increases the affinity of eIF2 for eIF2B, preventing nucleotide exchange on eIF2 by eIF2B. The reduction in TC levels brings about large-scale reductions in overall protein synthesis rates and translational activation of specific genes that causes wider reprogramming of gene expression in what is termed the integrated stress response (ISR) (Pavitt, 2018; Wek, 2018).

eIF2B is a large decameric protein complex comprised of two copies of each of five distinct subunits (α–ε), as revealed by mass spectrometry and structural studies, with a hexameric core comprised of two α, β, and δ subunits linked to two separate catalytic “arms,” each of γε heterodimers (Gordiyenko et al., 2014; Kashiwagi et al., 2016). Remarkably, eIF2 was shown to bind to two distinct binding sites on eIF2B with eIF2α binding at an interface formed either between eIF2Bα and δ or between eIF2Bβ and δ. These findings provided insight into the phospho-regulatory mechanism (Adomavicius et al., 2019; Gordiyenko et al., 2019; Kashiwagi et al., 2019; Kenner et al., 2019). The discovery of a chemical inhibitor of the ISR, ISRIB, that binds directly to eIF2B across the axis of symmetry at the (βδ)_2_ core and allosterically modulates the sensitivity of eIF2B to eIF2α phosphorylation (Schoof et al., 2021; Sidrauski et al., 2013; Zyryanova et al., 2021) has led to renewed interest in how eIF2B coordinates cellular activity, particularly as ISRIB, can enhance aspects of neurological activity including memory in several contexts (Costa-Mattioli and Walter, 2020).

ISRIB is not the only potential ligand modifier of eIF2B activity that has been identified. Trazodone hydrochloride and dibenzoylmethane were both found to act as ISR inhibitors that likely target eIF2B and showed therapeutic potential in mouse models of neurodegenerative disorders, where eIF2 phosphorylation and the ISR are elevated (Halliday et al., 2017). Accordingly, an eIF2B targeting agonist entered human trials in 2020. In addition, eIF2B may bind naturally occurring ligands. eIF2Bαβδ subunits show structural similarity to ribose bisphosphate isomerases, and Kuhle and colleagues established that both AMP and GMP can bind to eIF2Bα (Kuhle et al., 2015). The role of ligand binding to eIF2Bα was very recently expanded to show that sugar phosphates including fructose-6-phosphate can bind eIF2Bα to enhance its GEF activity, demonstrating that eIF2B activity can be modified by naturally occurring ligands other than phosphorylated eIF2 (Hao et al., 2021). It was previously shown that GTP can directly bind to purified yeast eIF2B, with an apparent *K*_*d*_ of 1 μM (Nika et al., 2000). Sequence and structural similarity between hexose sugar-nucleotide pyrophosphorylase (HNP) enzymes and the eIF2Bγε subunits forming the GEF catalytic arms suggested that GTP may bind here. Even though mutagenesis of conserved residues in eIF2Bε failed to indicate a role for nucleotide binding (Reid et al., 2012), mass spectrometry indicated that GTP was bound to eIF2Bγ when eIF2B was purified from yeast (Gordiyenko et al., 2014). To explore the role of GTP or other nucleotide binding to eIF2B we have here combined investigations with purified proteins, yeast genetics, and GEF assays to show that both ATP and GTP can bind to eIF2Bγ and that GTP, but not ATP-binding, can enhance the rate of nucleotide exchange of GDP bound to eIF2. Some kinetic reaction schemes for eIF2B include a sequential mechanism where GTP bound to eIF2B as a prerequisite for release of GDP from eIF2 (Manchester, 2001; Price and Proud, 1994), but as recently discussed, this appears unlikely (Bogorad et al., 2018). We propose these data are consistent with GTP binding to eIF2Bγ serving an allosteric regulatory role, enabling eIF2B to sense local energy charge rather than being necessary for GEF action *per se*. These and other findings suggest that eIF2B complexity enables it to bind a range of ligands to fine-tune its activity.

## Results and discussion

### GTP binding by eIF2B activates its GEF activity

**Figure 1:**
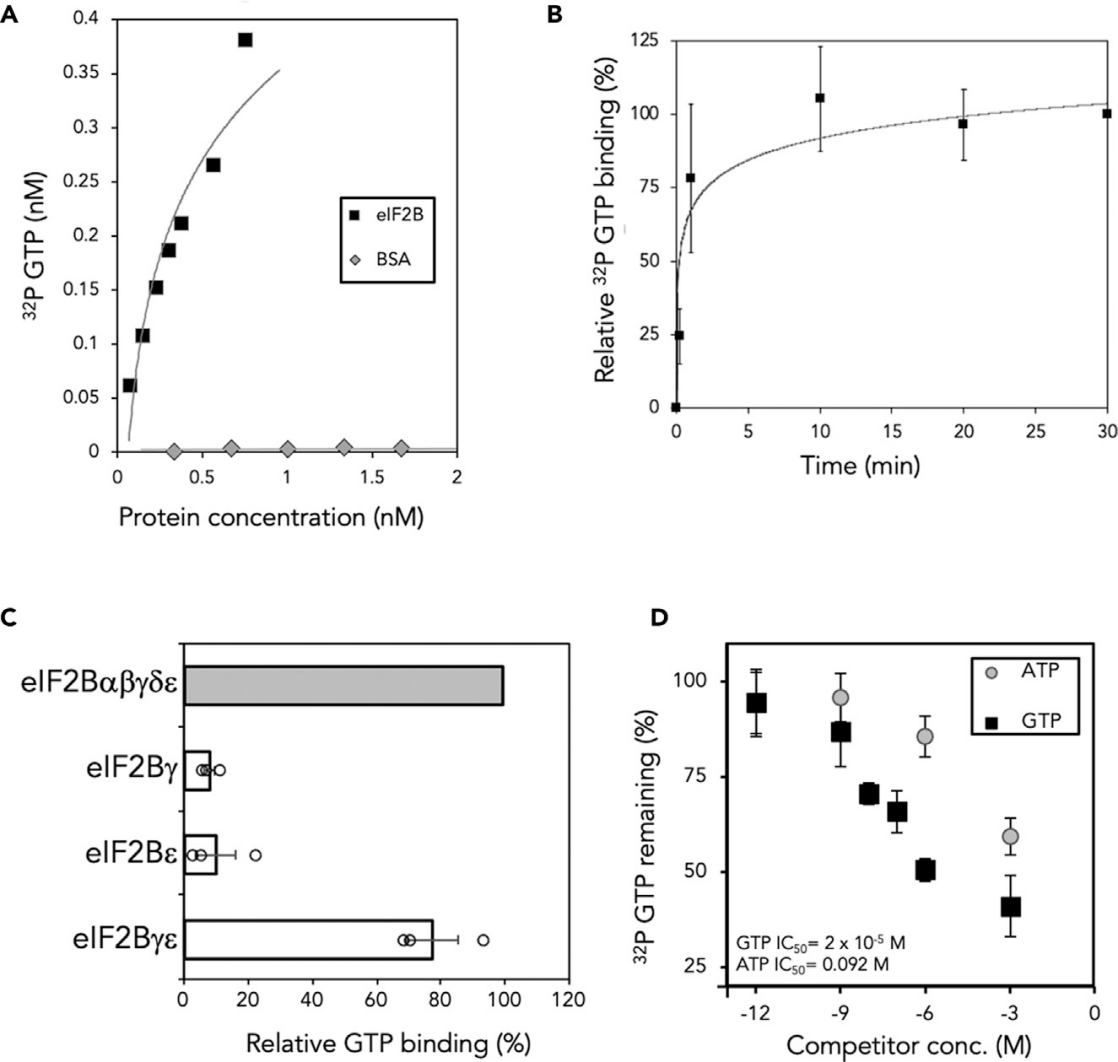
eIF2B binds GTP (A) Purified eIF2B (Figure S1A) binds radiolabeled GTP in a concentration-dependent manner. Increasing concentrations of eIF2B bind ^32P^GTP; BSA was used as a control. (B) GTP binding by eIF2B increases over time. Error is shown as standard error of the mean (s.e.m). (C) Both eIF2Bγ and ε are required for binding of GTP. GTP binding is expressed relative to eIF2B complexes (12 μmol of each protein used), and error is shown as s.e.m of ≥3 technical replicates. (D) Competition binding assays with indicated excess concentrations of unlabeled ATP or GTP. Error as panel B. See also Figure S1.

In accord with previous reports (Gordiyenko et al., 2014; Nika et al., 2000), we found using a nitrocellulose filter binding assay that ^32^P-GTP binds to eIF2B purified from yeast cells in a concentration- and time-dependent manner (Figures 1A, 1B, and S1A). As eIF2 is a classic GTP binding protein and the major partner of eIF2B, we used 500 mM KCl in our purification buffers, which ensures removal of all detectible traces of eIF2 from our eIF2B preparations (Adomavicius et al., 2019; Gordiyenko et al., 2014; Mohammad-Qureshi et al., 2007b). As fluorescent GTP analogs can bind eIF2 (Jennings et al., 2017), we evaluated whether they would also interact with eIF2B and detected no eIF2B-dependent change in fluorescent signal for all those tested, suggesting they do not bind (not shown). We interpreted these data as indicating that the position of the fluorescent label attachment allows binding to classic GTP binding proteins, but not to eIF2B, which lacks the G protein signature elements. We therefore used radiolabeled or unlabeled nucleotides for our eIF2B studies and BODIPY-labeled GDP for GEF assays with eIF2. Mass spectrometry experiments previously implicated eIF2Bγ as GTP-binding subunit (Gordiyenko et al., 2014), but purified isolated eIF2Bγ or its binding partner eIF2Bε, both independently failed to bind ^32^P-GTP. In contrast, a purified eIF2Bγε sub-complex did bind ^32^P-GTP, comparable with the levels seen for full eIF2B complexes (Figure 1C). This result is consistent with the idea that eIF2Bε stabilizes a conformation of eIF2Bγ (or vice versa) that permits nucleotide binding. A similar interpretation was invoked for studies that showed eIF2Bγε binds better to eIF2 and is a more active GEF than eIF2Bε alone (Pavitt et al., 1998).

To explore further nucleotide binding to eIF2B, we used a competition assay with a series of unlabeled nucleotides. These assays showed that GTP was a more effective competitor than ATP or GDP but that excess of both adenine and guanine nucleotides were able to compete off ^32^P-GTP, suggesting eIF2B can bind both ATP and GTP, but with a preference for GTP (Figures 1D and S1B).

**Figure 2:**
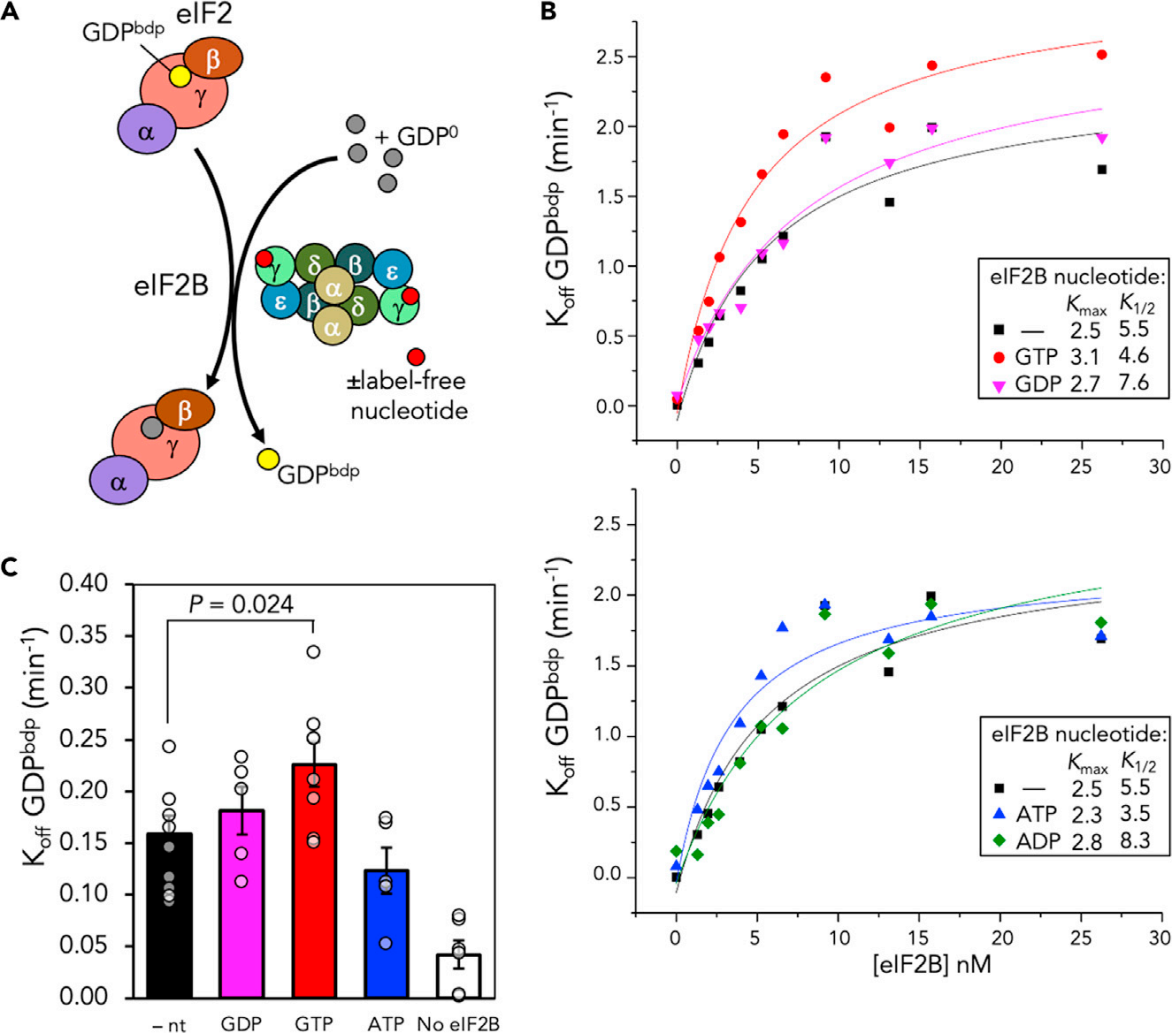
GTP binding enhances the rate of nucleotide exchange (A) Diagrammatic representation of assay monitoring release of BODIPY GDP from eIF2 in the presence of excess unlabeled GDP. (B) Only GTP boosts K_off_ of eIF2B. GDP dissociation assays were performed with eIF2B preincubated with increasing [GTP], [GDP] (top), [ATP], or [ADP] (bottom); *K*_max_ and *K*_1/2_ were calculated from the line of best fit, as described in the STAR Methods. (C) GTP boosts K_off_ at physiological [eIF2:eIF2B] 8:1. n = 3 ± s.e.m, one-tailed t test. See also Figure S2.

We next evaluated the consequence of nucleotide binding to eIF2B for its GEF activity. In our standard GEF assay we preformed eIF2-GDP complexes with the fluorescent BODIPY-labeled GDP (GDP^bdp^) and incubated these complexes with excess unlabeled GDP and a range of concentrations of eIF2B to determine the rate of GDP^bdp^ release and reaction kinetic parameters (Figures 2A and S2A). As expected from previous results (Jennings et al., 2016, 2017), increasing concentrations of eIF2B promote faster release of GDP from eIF2 (Figure 2B). Remarkably, we found that preincubating purified eIF2B with unlabeled GTP boosted the rate of GEF activity in this assay (Figures 2B and 2C), whereas preincubation with all other nucleotides tested did not. GTP both increased the maximum rate of GTP release (*K*_*max*_) from 2.5 to 3.1 min^−1^ and reduced the calculated *K*_*1/2*_ from 5.5 to 4.2 nM eIF2B (Figure 2B). ATP reduced the *K*_*1/2*_, but at a physiological ratio of eIF2:eIF2B of 8, only GTP significantly boosted the off-rate of GDP^bdp^ by approximately 50% (Figure 2C). Importantly the rate of spontaneous eIF2B-independent GDP^bdp^ release from eIF2 was not impacted by the presence of additional unlabeled nucleotides (Figure S2B). Together the data shown in Figures 1 and 2 imply that GTP binding to the eIF2Bγε arm can boost its GEF activity toward eIF2-GDP.

### Structural modeling suggests GTP can bind a surface pocket on eIF2Bγ

**Figure 3:**
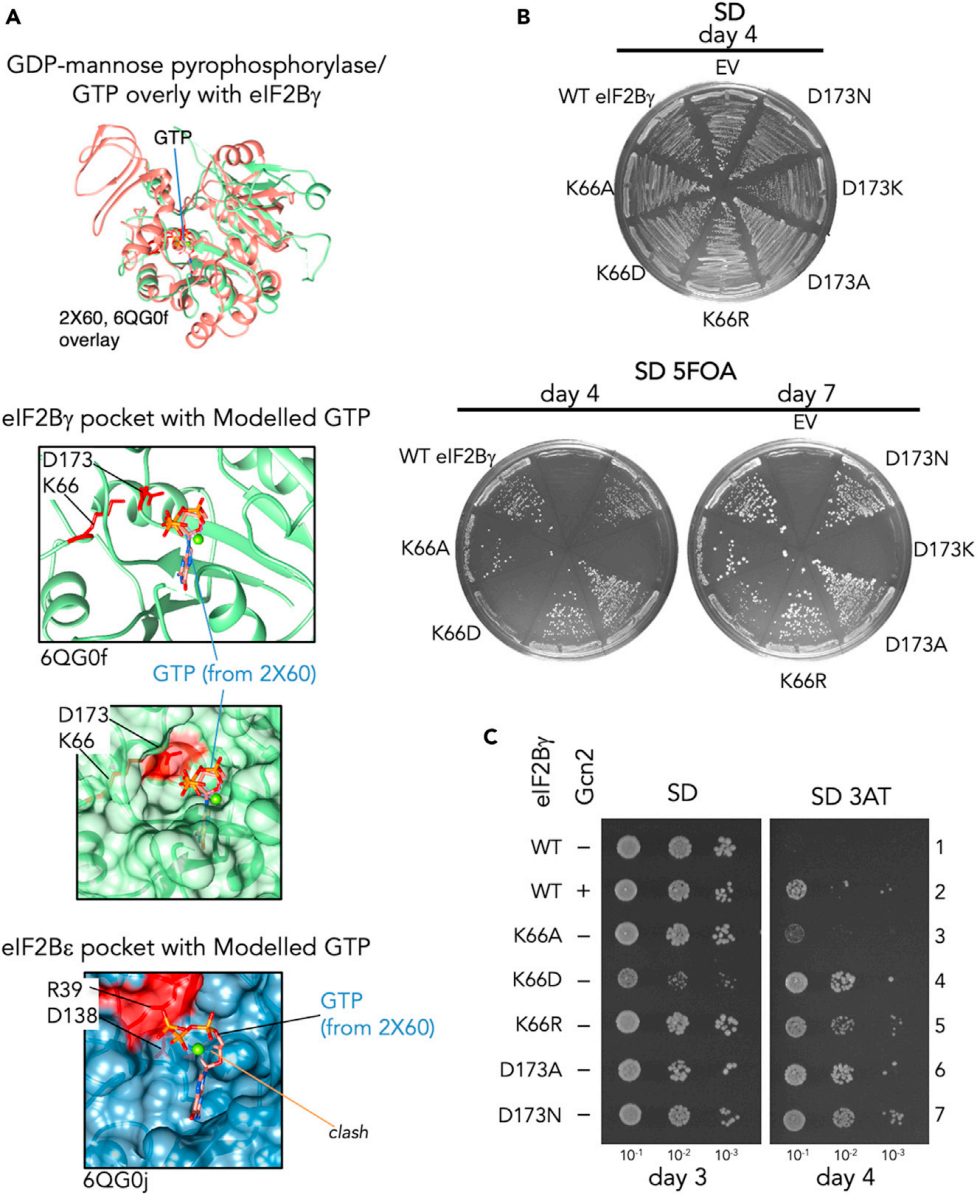
Mutations in the potential GTP-binding pocket of eIF2Bγ impact cell growth and stress responses (A) Structural alignment of GDP-mannose pyrophosphorylase bound to GTP (2X60, salmon) with yeast eIF2Bγ (6QG0f, green) or eIF2Bε (6QG0j, blue) showing modeled contacts with GTP (red highlighted residues). (Top) Overview; (middle) GTP pocket detail showing eIF2Bγ and GTP only; and (bottom) protein surface rendering. Structural analysis and images used UCSFChimera software version 1.15 (Pettersen et al., 2004). (B) Plasmid shuffle removing the *URA3 GCD1* plasmid using 5FOA leaving the mutated *GCD1* as the sole version of eIF2Bγ. SD plates without 5FOA are shown for control. (C) Growth of strains ±3AT. See also Figures S3 and S4.

Recent cryo-electron microscopy (cryoEM) studies have provided insight into eIF2B architecture and its interactions with eIF2 (Adomavicius et al., 2019; Gordiyenko et al., 2019; Kashiwagi et al., 2019; Kenner et al., 2019). However, the eIF2Bγ subunits in each structure are the least well-resolved. Nevertheless, as predicted initially based on sequence similarity (Koonin, 1995), both the eIF2Bε and γ subunits display structural homology to members of the HNP enzyme family that bind specific sugars and nucleotides. For example, ADP-glucose pyrophosphorylase (Pdb: 1YP3, Figure S3A) binds ATP and glucose to form ADP-glucose (Jin et al., 2005), whereas GDP-mannose pyrophosphorylase (Pdb 2X60; Figure S3B) binds GTP and mannose (Pelissier et al., 2010). Aligning the eIF2Bγ, eIF2Bε, and eIF2B pyrophosphorylase-like (PL) domain sequences to these HNP enzymes shows that key charged nucleotide-binding residues (e.g. K25 and D109 in 2X60) are conserved in eIF2Bγ (e.g. K66 and D173 in yeast eIF2Bγ), whereas the conserved lysine is typically substituted with arginine in eIF2Bε (Figure S3C). Aligning the structures placed these conserved residues in overlapping positions (Figure S3D) and enabled modeling of GTP into a deep pocket within yeast eIF2Bγ (Figures 3A and S3E). In contrast, eIF2Bε does not possess a deep surface pocket and the modeled nucleotide clashes with the protein (Figures 3A bottom and S3F). This modeling supports the idea that nucleotide binding to a pocket in eIF2Bγ is plausible, but it should be noted that not all eIF2Bγ residues have been modeled and no clear density was observed in the structures that could be attributed to any nucleotide.

### Identification of a guanine-sensitive mutant on eIF2Bγ

To further characterize potential nucleotide binding, we took advantage of well-developed genetic tools available in yeast and mutated *GCD1*, the gene encoding eIF2Bγ, at K66 and D173. Plasmids bearing variants were introduced into a *gcd1Δ* strain bearing wild-type (WT) *GCD1* on a plasmid (Figure 3B, top). Cells were transferred to medium containing 5-fluoro-orotic acid (5FOA) to select for growth of cells bearing only the introduced variant. Charge reversal mutants at each place had severe growth impairment with D173K being inviable, and K66D was slow growing (Figure 3B, bottom). All other mutants grew well on standard medium (SD), but (except K66A) conferred Gcn2-independent growth on medium containing the His3 inhibitor 3-aminotriazole (3AT), which is a hallmark of eIF2B mutations with reduced activity (Figure 3C, compare rows 3–7 with 1) (Hinnebusch, 2005). Examining protein levels showed that D173K expression levels were very low, likely explaining its lethality (Figure S4A). Although the other mutants had modest reductions in eIF2Bγ expression levels (Figure S4A), they all formed intact eIF2B complexes that could be isolated by Flag-immune precipitation (Figure S4B).

**Figure 4:**
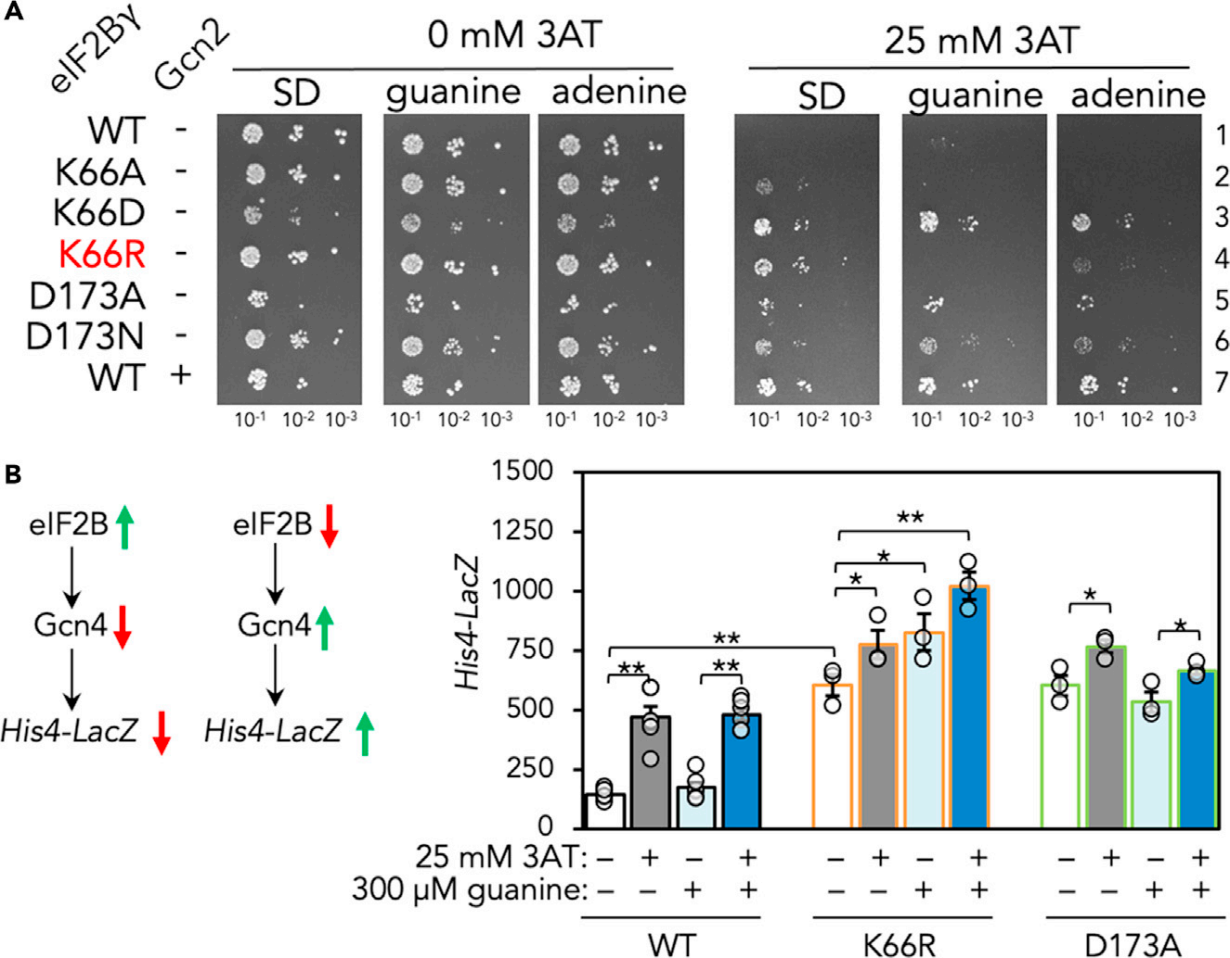
Guanine supplementation alters the yeast ISR for eIF2B(γK66R)-containing cells (A) Growth of eIF2Bγ mutants on selective medium containing or without guanine or adenine in the absence or presence of 3AT. Supplementation with guanine or adenine suppress the growth defect of K66R only. (B) (Left) Cartoons summarizing impact of changing eIF2B activity on Gcn4 and His4-lacZ. Green arrows show high activity red arrows show low activity. (Right) β-galactosidase activity of *His4-lacZ* for indicated mutants in *GCN2* cells with 3AT and/or guanine supplements shown. n = 3 ± s.e.m. t test, one-tailed ∗*P* < 0.05, ∗∗*P <* 0.01. See also Figure S5.

Mutations impairing guanine synthesis have been found to impact protein synthesis and *GCN4* translational control (Iglesias-Gato et al., 2011) (see below). So next we investigated whether supplementing growth medium with excess guanine or adenine would alter any of these phenotypes. We found that addition of excess guanine, and to a lesser extent adenine, specifically suppressed the Gcn2-independent growth of eIF2Bγ-K66R mutant cells on 3AT medium (Figures 4A row 4, and S5A). We made a matching set of mutations altering eIF2Bε R39 and D138, including the charge reversal changes, but these only showed phenotypes consistent with mild loss of function and were not affected by purine supplementation (Figure S5B). We also assessed in the same genetic assays previously described reduced function eIF2Bγ mutants as well as eIF2Bε mutants, equivalent to mutations causing vanishing white matter disease in humans. All these mutations in eIF2Bγ and ε exhibit Gcn2-independent growth on 3AT medium that was not reversed by guanine or adenine substitution (Figure S5). Thus eIF2B(γK66R) is the only mutant we analyzed that is differently sensitive to purine supplementation.

### eIF2B(γK66R) impacts Gcn4-mediated translational control of *HIS4*

In WT cells, growth on 3AT medium requires activation of Gcn2 kinase to phosphorylate eIF2α and inactivate eIF2B. This ISR activates translation of *GCN4* mRNA, which is normally silenced by inhibitory upstream open-reading frames in its 5′ leader sequence. Gcn4, a transcriptional activator of amino acid biosynthesis enzymes, enhances expression of amino acid biosynthetic enzyme mRNAs including *HIS4* (Figure 4A). Our yeast cells contain a genomically integrated *HIS4-lacZ* reporter of this response, so we monitored β-galactosidase activity in WT cells and those with K66R and D173A substitutions. As expected, WT *HIS4-LacZ* expression was 3AT-dependent. In contrast, K66R and D173R mutants expressed constitutively high levels of *HIS4*, consistent with their Gcn2-independent growth on 3AT medium (Figure 4B). Guanine did not alter the *HIS4* expression patterns of WT or D173A mutants in agreement with their growth on 3AT medium but boosted *HIS4* expression even higher in K66R mutant cells (Figure 4B). The K66R mutant responds to the elevated guanine levels, although in an opposite way to that, which might have been anticipated from the guanine-sensitive 3AT growth phenotype. Together the data suggest that excess purine supplementation impairs further the eIF2B activity of the K66R mutant, specifically diminishing growth on 3AT medium containing guanine or adenine.

### eIF2B(γK66R)–eIF2 interaction is GTP sensitive

**Figure 5:**
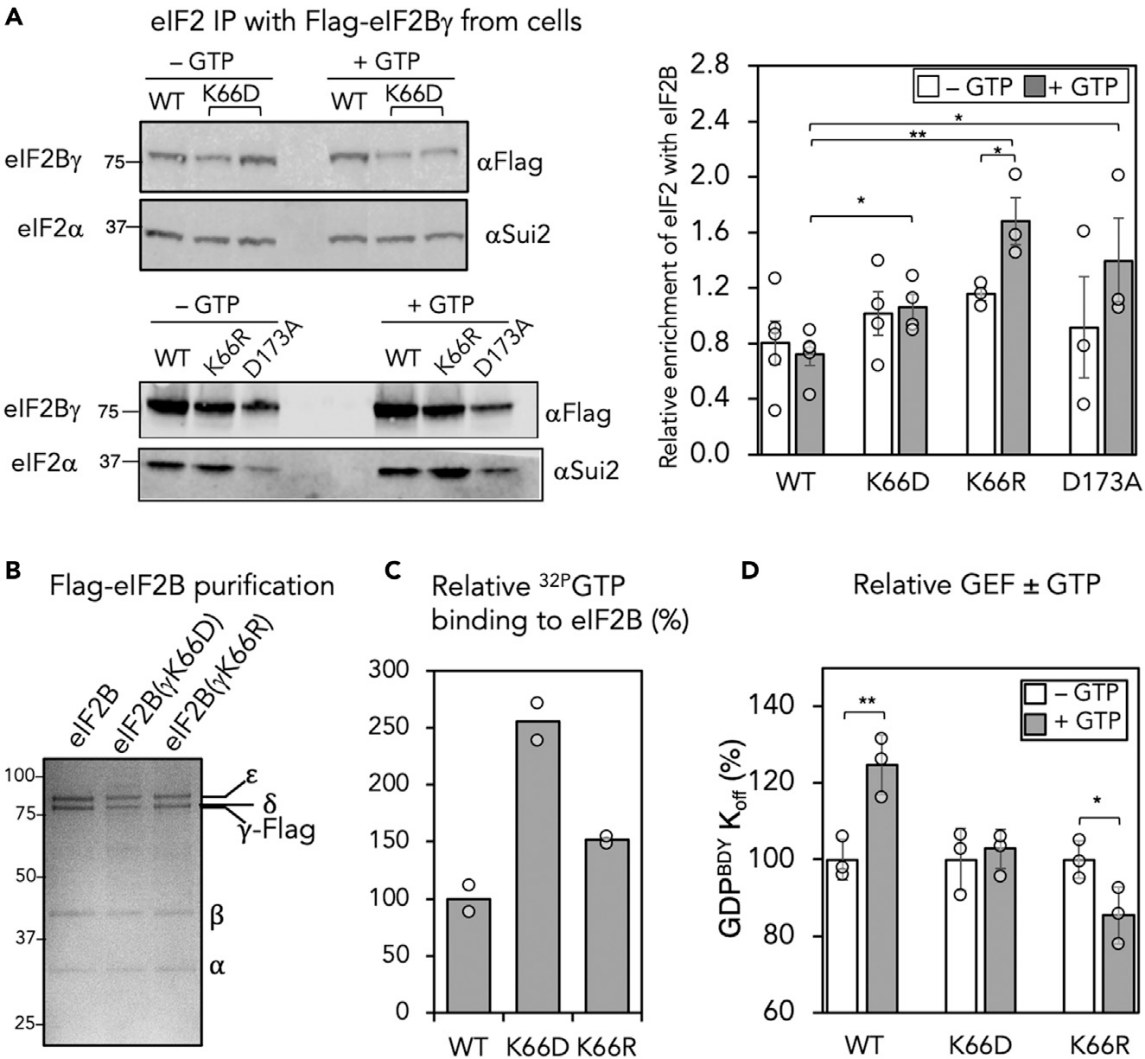
eIF2B(γK66) mutants impact eIF2B responses to GTP (A) Co-IP of eIF2 with flag-eIF2B ± 1 mM GTP from soluble protein cell extracts performed and quantification of relative eIF2 enrichment ±s.e.m. (n = 3). t test, one-tailed, ∗p < 0.05, ∗∗p *<* 0.01. (B) Coomassie-blue-stained gel of Flag affinity purified eIF2B complexes (1 μg). (C) Binding of GTP to 12 μmol purified eIF2B proteins (n = 2) (D) Effect of GTP on eIF2B GEF activity. Activity of 0.5 μg each protein—GTP is normalized to 100% to show the impact of prebinding GTP (n = 3). t test, one-tailed, ∗p < 0.05, ∗∗p *<* 0.01. See also Data S1.

To examine further the impact of the K66R mutation we performed Flag immunoprecipitation of the K66R mutant from yeast cells in our standard low-salt buffer and in the same buffer containing 1 mM GTP. GTP significantly enhanced recovery of eIF2 bound to eIF2B(γK66R) but did not alter binding of WT eIF2B or the other mutants tested (Figure 5A). This is consistent with the idea that the affinity of this mutant for eIF2 is sensitive to GTP concentrations. Tighter binding of eIF2 to eIF2B is known to be inhibitory to eIF2B GEF activity when eIF2 is phosphorylated (Jennings et al., 2017; Krishnamoorthy et al., 2001). Here, eIF2 binding by eIF2B(γK66R) was enhanced by GTP. Finally, to test directly the impact of the K66R mutation on GTP binding and GEF activity we purified eIF2B(γK66R) and eIF2B(γK66D) mutants (Figure 5B). Both mutants bound more ^32P^GTP than WT eIF2B (Figure 5C); however, prebinding of GTP only stimulated the activity of WT eIF2B and not either mutant, and the K66R mutant activity was impaired by excess GTP (Figure 5D), consistent with our *in vivo* results (Figure 4). Taken together, the data are consistent with an explanation that GTP binding to the eIF2Bγ HNP domain can enhance the rate of GDP release from eIF2, whereas specific mutation at γK66 prevents this occurring. The K66R results are consistent with GTP impairing eIF2 dissociation from eIF2B(γK66), such that more eIF2 is retained bound to eIF2B. This would interfere with eIF2 engagement with the preinitiation complex, impairing translation and cell growth. In contrast, GTP is able to enhance rates of wild-type eIF2B GEF activity, without affecting the stability of the overall eIF2-eIF2B interaction (Figures 5A and 5D).

**Figure 6:**
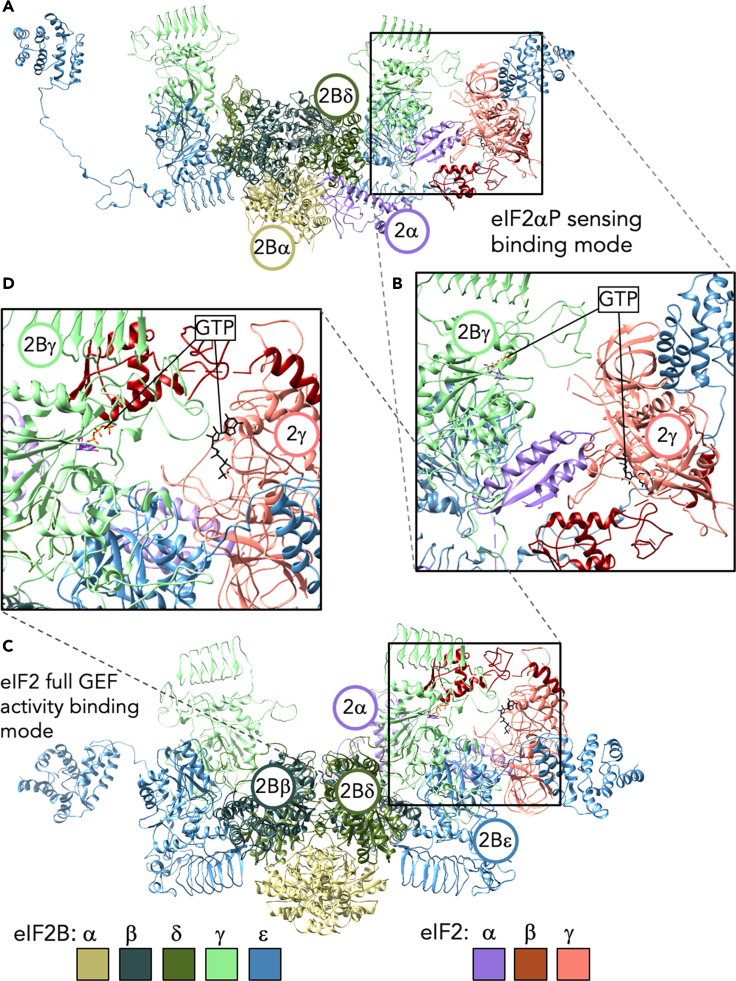
Structural models of GTP binding to eIF2 and eIF2B during complex interactions (A and B) Model of eIF2 bound to eIF2B in eIF2αP sensing mode and (B) detail of potential GTP-binding sites. GDPCP (GTP analog) modeled from eIF2 ternary complex (3JAP) added in black to eIF2γ (salmon) and GTP from GDP-mannose pyrophosphorylase (2X60) added in standard atom colors to eIF2Bγ (light green). (C) As panel A but depicting eIF2 bound engaging fully with eIF2Bepsilon. (D) Zoomed detail of modeled nucleotide binding, as described for panel B.

As indicated in the introduction, the role of potential GTP binding to eIF2B has been discussed over many years, with one model proposing direct transfer of GTP from eIF2B to eIF2γ as a mechanism of exchange. As we and others have argued previously, eIF2B does not require GTP to perform nucleotide release from eIF2–GDP *in vitro*. In its minimal form the eIF2Bε GEF domain can suffice to promote GDP release (de Almeida et al., 2013; Gomez et al., 2002). The yeast eIF2Bγε sub-complex has full GEF activity *in vitro*, which is at least 10-fold greater than eIF2Bε alone, although additional subunits are required in cells as eIF2Bβ and δ are encoded by essential genes and eIF2Bα is needed for the yeast ISR. When GTP concentration is high, local free GTP levels in the cytoplasm should facilitate efficient nucleotide exchange (see Bogorad et al. [2018] for a recent in-depth discussion of the thermodynamics of the interactions). However, the recent eIF2-eIF2B co-structures do place the GTP-binding surfaces of eIF2Bγ and eIF2γ subunits both adjacent to and facing each other (Adomavicius et al., 2019; Gordiyenko et al., 2019; Kashiwagi et al., 2019; Kenner et al., 2019). We therefore used molecular threading (Waterhouse et al., 2018) to generate more complete models of the yeast eIF2–eIF2B complexes with eIF2 bound at both “eIF2(αP)-sensing” interface and the “full-GEF activity” interface and then modeled GTP into the binding pockets on both factors (Figures 6A–6D). Although facing each other, the nucleotide-binding pockets in each protein are approximately 45 Å apart, the same distance in both modes of eIF2 binding. Although speculative, it seems plausible that GTP, or a related compound, binding to eIF2Bγ may cause modest local rearrangements that enhance the rate of exchange of the nucleotide bound to eIF2γ with free nucleotide in the cytoplasm. We postulate this would sensitize eIF2B to local nucleotide levels and help to fine-tune protein synthesis and the ISR to fluctuations in purine nucleotide levels.

Prior studies have shown that during exponential growth in yeast both the GTP:GDP and ATP:ADP ratios are ∼4–5:1. However, as glucose (the preferred carbon source) becomes limited in the culture both ratios drop to around 1:1, with the GTP:GTP ratio falling faster than ATP:ADP (Rudoni et al., 2001). Conversely adding glucose to trehalose grown cultures was found to both reverse this trend and boost the total guanine nucleotide pool. Within 10 min of adding glucose, the GTP:GDP ratio was restored to ∼4.5:1, and the total guanine nucleotide pool level approximately doubled (Walther et al., 2010). As GTP is the main energy source for protein synthesis, with GTP hydrolysis contributing to the function of G proteins during initiation (eIF2 and eIF5B), every elongation cycle (eEF1a and eEF2), and to termination (eRF3), using GTP levels to modulate eIF2B and hence initiation rates early in the translation cycle may help ensure cells do not overcommit resources.

It was recently demonstrated that eIF2Bα can bind sugar phosphates to a different regulatory pocket, which also enhanced eIF2B activity (Hao et al., 2021; Kuhle et al., 2015). Hence eIF2B has at least 2 pockets to which cell metabolites can bind and influence its activity. In addition, a third pocket formed at the interface between eIF2Bβ and δ dimers binds the synthetic ISR modifier ISRIB (Schoof et al., 2021; Sidrauski et al., 2013; Zyryanova et al., 2021). It is not yet known if a natural substrate can also bind within the ISRIB-binding (βδ)_2_ pocket. The equivalent region of yeast eIF2B has a smaller pocket than that observed for human eIF2B, and ISRIB cannot be modeled here without a steric clash (not shown). Prior work, especially from the Proud group, identified that GSK3 can phosphorylate eIF2Bε to modulate GEF activity (Wang et al., 2001). Together these studies suggest that every subunit of eIF2B has the potential to be modified by distinct inputs that combine to modulate GEF activity, consistent with eIF2B being an important regulatory hub that can sense a wide variety of inputs directly, in addition to the well-described phosphorylation of its substrate eIF2 in the ISR. Figuring out the relative importance of each under differing conditions is the next task.

### Limitations of the study

Our findings reported here provide new insights about the interplay between nucleotides and the activity of eIF2B, a key guanine nucleotide exchange factor in the ISR. However, our study uses *Saccharomyces* cerevisiae as a model organism so it will be important to assess whether mammalian/human eIF2B is also regulated in a similar way. A technical limit to radiolabeled GTP binding assays with purified eIF2B proteins is that we cannot completely exclude the possibility that there remains some small contamination with another GTP-binding factor. Given interest in modulating eIF2B activity in humans will also be important to assess other chemicals whose structure is related to GTP. Important molecular details about the interplay of GTP binding to eIF2B and its partner eIF2 remain to be studied.

## STAR★Methods

### Key resources table

**Table:** 

REAGENT or RESOURCE	SOURCE	IDENTIFIER
**Antibodies**		

Anti-Flag M2 magnetic beads	Sigma-Aldrich	M8823
Anti-Flag M2 mouse	Sigma-Aldrich	P2983, RRID:AB_439685
Anti-Flag M2 affinity gel	Sigma-Aldrich	A2220
Anti-Sui2 (rabbit)	Dr T. Dever, NIH	
Anti-Gcn3	Dr A. Hinnebusch, NIH	
Anti-Gcd7	Dr A. Hinnebusch, NIH	
Anti-Gcd6	Dr A. Hinnebusch, NIH	
Anti-Gcd2	Dr A. Hinnebusch, NIH	
Anti-Tef1	Dr C. Grant, Univ. Manchester UK	
IRDye® 800CW goat anti-rabbit IgG	LI-COR Biosciences	926-32211, RRID:AB_621843
IRDye® 680RD goat anti-mouse IgG	LI-COR Biosciences	925-68070, RRID:AB_2651128

**Bacterial and virus strains**		

*E. coli* XL1-Blue Supercompetent Cells	Agilent	200523

**Chemicals, peptides, and recombinant proteins**		

Complete Protease Inhibitor mini tablets, EDTA free	Roche	11836170001
Yeast Nitrogen Base Without Amino Acids	Formedium	CYN0402
SC, Double Drop-Out -Leu, -Ura	Formedium	DSCK1017
5-Fluoro Orotic Acid Monohydrate	Formedium	5FOA05
3-Amino-1,2,4-triazole	Sigma-Aldrich	A8056
Adenine	Sigma-Aldrich	A8626
Guanine	Sigma-Aldrich	G11950
Guanosine 5′-triphosphate sodium salt hydrate	Sigma-Aldrich	G8877
Guanosine 5′-diphosphate sodium salt	Sigma-Aldrich	G7127
Adenosine 5′-diphosphate sodium salt	Sigma-Aldrich	A2754
Guanosine 5'-Diphosphate, BODIPY™ FL 2'-(or-3')-O-(N-(2-Aminoethyl) Urethane), Bis (Triethylammonium) Salt)	ThermoFisher	G22360
GTP, [α-^32^P]- 3000Ci/mmol 10mCi/ml	PerkinElmer	BLU006H250UC
Ultima Gold F	PerkinElmer	6013179

**Critical commercial assays**		

QuikChange II site direct mutagenesis	Agilent	200523
NiNTA agarose	Qiagen	30210
HiTrap heparin	Cytiva	17-0407-01
HiTrap Q	Cytiva	17-1153-01

**Experimental models: Organisms/strains**		

*Saccharomyces cerevisiae* strains derived from S288c	Lab collection	see Table S1

**Oligonucleotides**		

GCGCTTGCCCGCGGCTCTTTTGCCCATCGGTAATAG	Sigma-Aldrich	K66A F
GCAAAAGAGCCGCGGGCAAGCGCGTTGAATGTTGC	Sigma-Aldrich	K66A R
GCGCTTGCCCGACGCTCTTTTGCCCATCGGTAATAG	Sigma-Aldrich	K66D F
GCAAAAGAGCGTCGGGCAAGCGCGTTGAATGTTGC	Sigma-Aldrich	K66D R
GCGCTTGCCCAGGGCTCTTTTGCCCATCGGTAATAG	Sigma-Aldrich	K66R F
GCAAAAGAGCCCTGGGCAAGCGCGTTGAATGTTGC	Sigma-Aldrich	K66R R
CTTGCCCTGCGCATTTGTCACAGATATACCTCCACAAGTC	Sigma-Aldrich	D173A F
CTGTGACAAATGCGCAGGGCAAGATTACAAAATCGCCATTG	Sigma-Aldrich	D173A R
CTTGCCCTGTAAGTTTGTCACAGATATACCTCCACAAGTC	Sigma-Aldrich	D173K F
CTGTGACAAACTTACAGGGCAAGATTACAAAATCGCCATTG	Sigma-Aldrich	D173K R
CTTGCCCTGTAATTTTGTCACAGATATACCTCCACAAGTC	Sigma-Aldrich	D173N F
CTGTGACAAAATTACAGGGCAAGATTACAAAATCGCCATTG	Sigma-Aldrich	D173N R
GACAGACTCTTATGAAACTAAATTTATGCCAC	Sigma-Aldrich	R39K F
CAGCTGTCAGTGGCATAAATTTAGTTTCATAAG	Sigma-Aldrich	R39K R
GACAGACTCTTATGAAAACTGATTTTATGCCAC	Sigma-Aldrich	R39D F
CAGCTGTCAGTGGCATAAAATCAGTTTCATAAG	Sigma-Aldrich	R39D R
GACAGACTCTTATGAAACTGAATTTATGCCAC	Sigma-Aldrich	R39E F
CAGCTGTCAGTGGCATAAATTCAGTTTCATAAG	Sigma-Aldrich	R39E R
GATTTTATTTTAGTCAGTGGTAATGTATTGACTAAC	Sigma-Aldrich	D138N F
GCTGAAATCGATGTTAGTCAATACATTACCACTGAC	Sigma-Aldrich	D138N R
GATTTTATTTTAGTCAGTGGTAAAGTATTGACTAAC	Sigma-Aldrich	D138K F
GCTGAAATCGATGTTAGTCAATACTTTACCACTGAC	Sigma-Aldrich	D138K R

**Recombinant DNA**		

Yeast expression plasmids	This study or cited references	See Table S2

**Software and algorithms**		

UCSF Chimera 1.15		(Pettersen et al., 2004)
homology modelling server SWISS-MODEL		(Waterhouse et al., 2018)
PISA		(Krissinel and Henrick, 2007)
FluorEssence	Horiba Scientific	
ImageStudio	Li-Cor BioSciences	

### Resource availability

#### Lead contact

Requests and information for reagents and resources will be fulfilled by Dr Graham Pavitt (graham.pavitt@manchester.ac.uk).

#### Materials availability

All stable reagents from this study are available from the lead contact.

### Experimental model and subject details

#### Strain construction

Yeast strains used in this study are listed in Table S1 and plasmids in Table S2.

Yeast cells were grown at 30°C.

#### Media preparation

All solid and liquid media have been prepared according to standard lab protocols. Standard synthetic complete media containing 2% glucose, but lacking nutritional supplements required for plasmid were used throughout (Adams et al., 1998). Where required, 3AT concentration was 25 mM, and adenine and guanine were added at the concentrations indicated in the figures.

### Method details

#### Site-directed mutagenesis

Mutations were introduced into plasmids pAV1265, pAV1418 and pAV1413 with the use of designed primers (key resources table). Site-directed mutagenesis was carried out with the QuikChange site-directed mutagenesis kit (Agilent Technologies).

#### Protein purification

eIF2B was purified from yeast strain GP5949, using Flag affinity gel and a high salt buffer containing 1 M KCl to ensure purification away from eIF2 as previously described (Mohammad-Qureshi et al., 2007b). eIF2B with K66 mutated eIF2Bγ variants were similarly purified from strains GP7050 and GP7051. eIF2 was purified by successive chromatography steps of Nickel affinity (Qiagen), HiTrap heparin and HiTrap Q sepharose (Cytiva) from strain GP3511 as described (Jennings et al., 2017).

#### GDP dissociation assay

Fluorescent eIF2•BODIPY-GDP binary complex was formed by incubating apo-eIF2 with a two times excess of BODIPY-FL-GDP (Thermo Fisher Scientific) and incubation for 20 minutes at room temperature. Excess nucleotide was removed by passing through a G-50 Sephadex column (GE Healthcare). Labelling efficiency was calculated to exceed 90%. To measure GDP release, 20 nM eIF2•BODIPY-GDP was quickly mixed with 1 mM of unlabelled GDP (± eIF2B) in 180 μl of assay buffer (30 mM HEPES, 100 mM KCl, 10 mM MgCl_2_, pH 7.4) and fluorescence intensity was continuously measured using a Fluoromax-4 spectrophotometer (Horiba) (490 nm excitation, 509 nm emission, 0.1 second integration time). For assays where nucleotides were pre-bound to eIF2B 1 mM nucleotide was incubated with eIF2B at RT for 20 mins to allow nucleotide binding then made 10mM MgCl_2_ before eIF2B was added to the eIF2 reactions. Experimental data were fitted to exponential dissociation curves to determine the rate constants (*K*_*off*_) at each eIF2B concentration. *K*_*1/2*_ and *K*_*max*_ values were determined from curve fitting y = [(*K*_*max*_ × x)/(*K*_*1/2*_ + x)] + c. Although eIF2B is a decamer with 2 binding sites for eIF2, we calculated its molarity as a pentamer with one copy of each subunit and a single binding site for eIF2.

#### Radiolabelled nucleotide binding assays

Filter binding assay was performed by adapting a previous eIF2 GEF assay (Gomez et al., 2002). Briefly, purified eIF2B was mixed with 0.1 μl GTP [γ-^32^P] (3000 Ci/mmol) (Perkin Elmer) in a final volume of 160 μl (10 mM HEPES pH 7.4, 100 mM KCl, 10 mM MgCl_2_) in a glass test tube and incubated at room temperature for 20 min (or the given time). The binding reaction was stopped by applying the reaction to a 0.45 μM cellulose nitrate membrane filter (Whatman WCN 25 mm diameter circles) fitted within a vacuum filter manifold (Millipore) and washed twice with 2.5 ml of ice-cold buffer (10 mM HEPES pH 7.4, 100 mM KCl, 10 mM MgCl_2_). Membranes were dried, submerged in 5 ml of Ultima Gold F scintillation fluid (Perkin Elmer) and counted in a Tri-Carb 2100TR liquid scintillation analyzer for 1 minute. Included unlabelled competitor nucleotides were added at indicated concentrations to test off-rate of the bound radiolabelled nucleotide.

#### Flag immunoprecipitation from whole cell extracts

Cells were grown to A_600_=1 in synthetic complete medium lacking leucine, centrifuged to pellet the cells which were frozen in liquid nitrogen and lysed by grinding under liquid nitrogen in a freezer mill (6870, Spex SamplePrep). The resulting frozen powder was resuspended in Ip-buffer at 2 ml/g cell pellet [100 mM KCl; 25 mM HEPES pH7.6; 2 mM MgCl_2_, 10% Glycerol, Complete Protease Inhibitor mini tablets, EDTA free (Roche) ± 1 mM GTP] and clarified by centrifugation at 5,500 x g at 4°C for 5 min and the supernatant clarified a second time by centrifugation at 16,000 x g at 4°C for 20 min. Anti-Flag M2 magnetic beads (Sigma-Aldrich Cat# M8823, RRID:AB_2637089) were washed x3 in lysis buffer ±GTP and 20 μl was incubated with 0.5 mg cell extracts for one hour at 4°C. Supernatant was removed and the captured beads were washed x3 with Ip-buffer before elution with 2x Laemli sample buffer at 95°C for 10 minutes. Eluted samples were resolved by SDS-PAGE. Bound eIF2Bγ-FLAG and eIF2α was probed using M2 mouse (Sigma-Aldrich Cat# P2983, RRID:AB_439685, 1:500 dilution) and specific rabbit Sui2 antibodies (1:1000) and quantitative IR Western blot detection was performed using IRDye® 800CW goat anti-rabbit IgG (LI-COR Biosciences Cat# 926-32211, RRID:AB_621843) or IRDye® 680RD goat anti-mouse IgG (LI-COR Biosciences Cat# 925-68070, RRID:AB_2651128) with an Odyssey Fc imaging system (Li-Cor). Signals for individual proteins were normalised to the mean signal for that antibody across each blot before determining the ratio of eIF2:eIF2B. Errors reflect variations in signals between blots/ biological replicate samples.

#### β-galactosidase assays

Assays to measure activity of a *HIS4* promoter driven *LacZ* reporter integrated at *ura3-52* in *gcd1* strains were done exactly as previously described (Dever, 1997) from cultures grown in SCD-uracil-histidine. n=3-6. Standard Error of the mean is reported. Guanine (300 μM) was added throughout culture growth where indicated. 25 mM 3AT was added for 6 hours prior to cell harvest where indicated.

#### Computational modelling

Modelling and visualization used UCSF Chimera software version 1.15 (Pettersen et al., 2004). Multiple structures were aligned using the 'matchmaker' tool with the Needleman-Wunsch algorithm using BLOSUM-100 matrix and standard parameters: secondary structure fraction: 0.3, gap open (HH/SS/other) 18/18/6, extend 1. For molecular threading model making models of the yeast eIF2-eIF2B complexes with eIF2 bound at both eIF2(αP)-sensing interface (eIF2Bαδ) and the 'full-GEF activity' interface (eIF2Bβδ) were computed with the homology modelling server SWISS-MODEL (Waterhouse et al., 2018). The amino acid sequence of each of eIF2 and eIF2B subunit was submitted as input in automated mode to identify suitable templates based on sequence alignment and structural homology. The top-ranking templates were manually selected to build multiple models, which were then aligned to published eIF2-eIF2B structures using UCSF Chimera (Pettersen et al., 2004). Individual yeast subunits were threaded on 6I3M (Adomavicius et al., 2019), 6JLZ (Kashiwagi et al., 2019) and 3JAP (Llacer et al., 2015), and aligned on 6I3M to create the model of eIF2-eIF2B with eIF2 bound at eIF2Bαδ, whereas threading on 6K71, 6JLY (Kashiwagi et al., 2019)and 3JAP with alignment to 6K71 was performed for the complex with eIF2 bound at eIF2Bβδ. eIF2Bε catalytic domain (1PAQ) (Boesen et al., 2004) was modelled to maintain the same position and orientation relative to eIF2γ in both conformations and was connected to eIF2Bε decameric body through a flexible linker rigidly adjusted in the surrounding space. The models were evaluated based on overall fitting in the electron density maps, molecular clashes and energetic stability of the subunit-subunit interfaces assessed with the software PISA (Krissinel and Henrick, 2007).

### Quantification and statistical analysis

Quantification of immunoblot signals was with the Li-Cor Odyssey imager. Signals for individual proteins were normalised to the mean signal for that antibody across each blot before determining the ratio of eIF2:eIF2B. Errors reflect variations in signals between blots/ biological replicate samples. Statistical analyses of all data types used the T-test as detailed in the individual Figure legends.

## Data Availability

Data: All data reported in this paper will be shared by the lead contact upon request. Code: This paper does not report original code. Any additional information required to reanalyze the data reported in this paper is available from the lead contact upon request.

## Supporting citations

The following references appear in the Supplemental information: Bushman et al., 1993; Hill and Struhl, 1988; Jones, 1991; Mohammad-Qureshi et al., 2007a; Richardson et al., 2004; Sievers et al., 2011; Sikorski and Hieter, 1989; Wek et al., 1990; Yang and Hinnebusch, 1996.

## References

[bib1] Adams A., Gottschling D.E., Kaiser C.A., Stearns T. (1998).

[bib2] Adomavicius T., Guaita M., Zhou Y., Jennings M.D., Latif Z., Roseman A.M., Pavitt G.D. (2019). The structural basis of translational control by eIF2 phosphorylation. Nat. Commun..

[bib3] Boesen T., Mohammad S.S., Pavitt G.D., Andersen G.R. (2004). Structure of the catalytic fragment of translation initiation factor 2B and identification of a critically important catalytic residue. J. Biol. Chem..

[bib4] Bogorad A.M., Lin K.Y., Marintchev A. (2018). eIF2B mechanisms of action and regulation: a thermodynamic view. Biochemistry.

[bib5] Bushman J.L., Asuru A.I., Matts R.L., Hinnebusch A.G. (1993). Evidence that *GCD6* and *GCD7*, translational regulators of *GCN4* are subunits of the guanine nucleotide exchange factor for eIF-2 in *Saccharomyces cerevisiae*. Mol. Cell. Biol..

[bib6] Costa-Mattioli M., Walter P. (2020). The integrated stress response: from mechanism to disease. Science.

[bib7] de Almeida R.A., Fogli A., Gaillard M., Scheper G.C., Boesflug-Tanguy O., Pavitt G.D. (2013). A yeast purification system for human translation initiation factors eIF2 and eIF2Bepsilon and their use in the diagnosis of CACH/VWM disease. PLoS One.

[bib8] Dever T.E. (1997). Using *GCN4* as a reporter of eIF2a phosphorylation and translational regulation in yeast. Methods.

[bib9] Gomez E., Mohammad S.S., Pavitt G.D. (2002). Characterization of the minimal catalytic domain within eIF2B: the guanine-nucleotide exchange factor for translation initiation. EMBO J..

[bib10] Gordiyenko Y., Llacer J.L., Ramakrishnan V. (2019). Structural basis for the inhibition of translation through eIF2alpha phosphorylation. Nat. Commun..

[bib11] Gordiyenko Y., Schmidt C., Jennings M.D., Matak-Vinkovic D., Pavitt G.D., Robinson C.V. (2014). eIF2B is a decameric guanine nucleotide exchange factor with a gamma2epsilon2 tetrameric core. Nat. Commun..

[bib12] Halliday M., Radford H., Zents K.A.M., Molloy C., Moreno J.A., Verity N.C., Smith E., Ortori C.A., Barrett D.A., Bushell M. (2017). Repurposed drugs targeting eIF2α-P-mediated translational repression prevent neurodegeneration in mice. Brain.

[bib13] Hao Q., Heo J.M., Nocek B.P., Hicks K.G., Stoll V.S., Remarcik C., Hackett S., LeBon L., Jain R., Eaton D. (2021). Sugar phosphate activation of the stress sensor eIF2B. Nat. Commun..

[bib14] Hill D.E., Struhl K. (1988). Molecular characterization of *GCD1*, a yeast gene required for general control of amino acid biosynthesis and cell-cycle initiation. Nucleic Acids Res..

[bib15] Hinnebusch A.G. (2005). Translational regulation of GCN4 and the general amino acid control of yeast. Annu. Rev. Microbiol..

[bib16] Hinnebusch A.G. (2014). The scanning mechanism of eukaryotic translation initiation. Annu. Rev. Biochem..

[bib17] Iglesias-Gato D., Martin-Marcos P., Santos M.A., Hinnebusch A.G., Tamame M. (2011). Guanine nucleotide pool imbalance impairs multiple steps of protein synthesis and disrupts GCN4 translational control in Saccharomyces cerevisiae. Genetics.

[bib18] Jennings M.D., Kershaw C.J., Adomavicius T., Pavitt G.D. (2017). Fail-safe control of translation initiation by dissociation of eIF2alpha phosphorylated ternary complexes. eLife.

[bib19] Jennings M.D., Kershaw C.J., White C., Hoyle D., Richardson J.P., Costello J.L., Donaldson I.J., Zhou Y., Pavitt G.D. (2016). eIF2beta is critical for eIF5-mediated GDP-dissociation inhibitor activity and translational control. Nucleic Acids Res..

[bib20] Jin X., Ballicora M.A., Preiss J., Geiger J.H. (2005). Crystal structure of potato tuber ADP-glucose pyrophosphorylase. EMBO J..

[bib21] Jones E.W. (1991). Tackling the protease problem in *Saccharomyces cerevisiae*. Methods Enzymol..

[bib22] Kashiwagi K., Takahashi M., Nishimoto M., Hiyama T.B., Higo T., Umehara T., Sakamoto K., Ito T., Yokoyama S. (2016). Crystal structure of eukaryotic translation initiation factor 2B. Nature.

[bib23] Kashiwagi K., Yokoyama T., Nishimoto M., Takahashi M., Sakamoto A., Yonemochi M., Shirouzu M., Ito T. (2019). Structural basis for eIF2B inhibition in integrated stress response. Science.

[bib24] Kenner L.R., Anand A.A., Nguyen H.C., Myasnikov A.G., Klose C.J., McGeever L.A., Tsai J.C., Miller-Vedam L.E., Walter P., Frost A. (2019). eIF2B-catalyzed nucleotide exchange and phosphoregulation by the integrated stress response. Science.

[bib25] Koonin E.V. (1995). Multidomain organization of eukaryotic guanine nucleotide exchange translation initiation factor eIF-2B subunits revealed by analysis of conserved sequence motifs. Protein Sci..

[bib26] Krishnamoorthy T., Pavitt G.D., Zhang F., Dever T.E., Hinnebusch A.G. (2001). Tight binding of the phosphorylated alpha subunit of initiation factor 2 (eIF2alpha) to the regulatory subunits of guanine nucleotide exchange factor eIF2B is required for inhibition of translation initiation. Mol. Cell Biol..

[bib27] Krissinel E., Henrick K. (2007). Inference of macromolecular assemblies from crystalline state. J. Mol. Biol..

[bib28] Kuhle B., Eulig N.K., Ficner R. (2015). Architecture of the eIF2B regulatory subcomplex and its implications for the regulation of guanine nucleotide exchange on eIF2. Nucleic Acids Res..

[bib29] Llacer J.L., Hussain T., Marler L., Aitken C.E., Thakur A., Lorsch J.R., Hinnebusch A.G., Ramakrishnan V. (2015). Conformational differences between open and closed states of the eukaryotic translation initiation complex. Mol. Cell.

[bib30] Manchester K.L. (2001). Catalysis of guanine nucleotide exchange on eIF2 by eIF2B: can it be both a substituted enzyme and a sequential mechanism?. Biochem. Biophys. Res. Commun..

[bib31] Merrick W.C., Pavitt G.D. (2018). Protein synthesis initiation in eukaryotic cells. Cold Spring Harb. Perspect. Biol..

[bib32] Mohammad-Qureshi S.S., Haddad R., Hemingway E.J., Richardson J.P., Pavitt G.D. (2007). Critical contacts between the eukaryotic initiation factor 2B (eIF2B) catalytic domain and both eIF2beta and -2gamma mediate guanine nucleotide exchange. Mol. Cell Biol..

[bib33] Mohammad-Qureshi S.S., Haddad R., Palmer K.S., Richardson J.P., Gomez E., Pavitt G.D. (2007). Purification of FLAG-tagged eukaryotic initiation factor 2B complexes, subcomplexes, and fragments from Saccharomyces cerevisiae. Methods Enzymol..

[bib34] Nika J., Yang W., Pavitt G.D., Hinnebusch A.G., Hannig E.M. (2000). Purification and kinetic analysis of eIF2B from Saccharomyces cerevisiae. J. Biol. Chem..

[bib35] Pavitt G.D. (2018). Regulation of translation initiation factor eIF2B at the hub of the integrated stress response. Wiley Interdiscip. Rev. RNA.

[bib36] Pavitt G.D., Ramaiah K.V., Kimball S.R., Hinnebusch A.G. (1998). eIF2 independently binds two distinct eIF2B subcomplexes that catalyze and regulate guanine-nucleotide exchange. Genes Dev..

[bib37] Pelissier M.C., Lesley S.A., Kuhn P., Bourne Y. (2010). Structural insights into the catalytic mechanism of bacterial guanosine-diphospho-D-mannose pyrophosphorylase and its regulation by divalent ions. J. Biol. Chem..

[bib38] Pettersen E.F., Goddard T.D., Huang C.C., Couch G.S., Greenblatt D.M., Meng E.C., Ferrin T.E. (2004). UCSF Chimera–a visualization system for exploratory research and analysis. J. Comput. Chem..

[bib39] Pochopien A.A., Beckert B., Kasvandik S., Berninghausen O., Beckmann R., Tenson T., Wilson D.N. (2021). Structure of Gcn1 bound to stalled and colliding 80S ribosomes. Proc. Natl. Acad. Sci. U S A.

[bib40] Price N., Proud C. (1994). The guanine nucleotide-exchange factor, eIF-2B. Biochimie.

[bib41] Reid P.J., Mohammad-Qureshi S.S., Pavitt G.D. (2012). Identification of intersubunit domain interactions within eukaryotic initiation factor (eIF) 2B, the nucleotide exchange factor for translation initiation. J. Biol. Chem..

[bib42] Richardson J.P., Mohammad S.S., Pavitt G.D. (2004). Mutations causing childhood ataxia with central nervous system hypomyelination reduce eukaryotic initiation factor 2B complex formation and activity. Mol. Cell. Biol..

[bib43] Rudoni S., Colombo S., Coccetti P., Martegani E. (2001). Role of guanine nucleotides in the regulation of the Ras/cAMP pathway in Saccharomyces cerevisiae. Biochim. Biophys. Acta..

[bib44] Schoof M., Boone M., Wang L., Lawrence R., Frost A., Walter P. (2021). eIF2B conformation and assembly state regulate the integrated stress response. eLife.

[bib45] Sidrauski C., Acosta-Alvear D., Khoutorsky A., Vedantham P., Hearn B.R., Li H., Gamache K., Gallagher C.M., Ang K.K., Wilson C. (2013). Pharmacological brake-release of mRNA translation enhances cognitive memory. eLife.

[bib46] Sievers F., Wilm A., Dineen D., Gibson T.J., Karplus K., Li W., Lopez R., McWilliam H., Remmert M., Soding J. (2011). Fast, scalable generation of high-quality protein multiple sequence alignments using Clustal Omega. Mol. Syst. Biol..

[bib47] Sikorski R.S., Hieter P. (1989). A system of shuttle vectors and yeast host strains designed for efficient manipulation of DNA in Saccharomyces cerevisiae. Genetics.

[bib48] Walther T., Novo M., Rossger K., Letisse F., Loret M.O., Portais J.C., Francois J.M. (2010). Control of ATP homeostasis during the respiro-fermentative transition in yeast. Mol. Syst. Biol..

[bib49] Wang X., Paulin F.E., Campbell L.E., Gomez E., O'Brien K., Morrice N., Proud C.G. (2001). Eukaryotic initiation factor 2B: identification of multiple phosphorylation sites in the epsilon-subunit and their functions in vivo. EMBO J..

[bib50] Waterhouse A., Bertoni M., Bienert S., Studer G., Tauriello G., Gumienny R., Heer F.T., de Beer T.A.P., Rempfer C., Bordoli L. (2018). SWISS-MODEL: homology modelling of protein structures and complexes. Nucleic Acids Res..

[bib51] Wek R.C. (2018). Role of eIF2alpha kinases in translational control and adaptation to cellular stress. Cold Spring Harb. Perspect. Biol..

[bib52] Wek R.C., Ramirez M., Jackson B.M., Hinnebusch A.G. (1990). Identification of positive-acting domains in GCN2 protein kinase required for translational activation of *GCN4* expression. Mol. Cell Biol..

[bib53] Yang W., Hinnebusch A.G. (1996). Identification of a regulatory subcomplex in the guanine nucleotide exchange factor eIF2B that mediates inhibition by phosphorylated eIF2. Mol. Cell Biol..

[bib54] Zyryanova A.F., Kashiwagi K., Rato C., Harding H.P., Crespillo-Casado A., Perera L.A., Sakamoto A., Nishimoto M., Yonemochi M., Shirouzu M. (2021). ISRIB blunts the integrated stress response by allosterically antagonising the inhibitory effect of phosphorylated eIF2 on eIF2B. Mol. Cell.

