# Supplementary material for: GTP binding to translation factor eIF2B stimulates its guanine nucleotide exchange activity
Source: iScience. 2021 Nov 14;24(12):103454. doi: 10.1016/j.isci.2021.103454 (PMC8633983; doi:10.1016/j.isci.2021.103454)

iScience, Volume *24*

Supplemental information

GTP binding to translation factor eIF2B stimulates its guanine nucleotide exchange activity

Christopher J. Kershaw, Martin D. Jennings, Francesco Cortopassi, Margherita Guaita, Hawra Al-Ghafli, and Graham D. Pavitt

Table S1. Yeast strains.
Related to STAR Methods.

Yeast strain	Genotype	Reference
GP3511	*MATα ura3-52 leu2-3 leu2-112 ino1 pep4::LEU2 sui2Δ gcn2Δ ura3-52::P-HIS4-LacZ* pAV1089 [hc his6-*GCD11 SUI2 SUI3 URA3* 2μ]	Pavitt et al., 1998
GP3750/H1905	*MATα leu2-3 leu2-112 ura3-52 ino1 gcd6Δ gcn2Δ::hisG Ura3-52::HIS4-lacZ* [*GCD6 CEN URA3*]	Bushman et al., 1993
GP3754	*MAT***a** *leu2-3 leu2-112 trp1-Δ63 ura3-52 GAL2+ gcd1::LEU2 Ura3-52::HIS4-lacZ gcn2Δ* [*GCD1 URA3 CEN*]	GP lab collection
GP3583/BJ1995	*MATα prb1-1122 pep4-3 leu2 trp1 ura3-52 gal2*	Jones, 1991
GP4228	*MATα leu2-3 leu2-112 ura3-52 ino1 gcd6Δ gcn2Δ::hisG Ura3-52::HIS4-lacZ* [*gcd6*(V57G) *CEN LEU2*] [*URA3 CEN*]	Richardson et al., 2004
GP4229	*MATα leu2-3 leu2-112 ura3-52 ino1 gcd6Δ gcn2Δ::hisG Ura3-52::HIS4-lacZ* [*gcd6*(I90F) *CEN LEU2*] [*URA3 CEN*]	Richardson et al., 2004
GP4230	*MATα leu2-3 leu2-112 ura3-52 ino1 gcd6Δ gcn2Δ::hisG Ura3-52::HIS4-lacZ* [*gcd6*(R284H) *CEN LEU2*] [*URA3 CEN*]	Richardson et al., 2004
GP4231	*MATα leu2-3 leu2-112 ura3-52 ino1 gcd6Δ gcn2Δ::hisG Ura3-52::HIS4-lacZ* [*gcd6*(R323P) *CEN LEU2*] [*URA3 CEN*]	Richardson et al., 2004
GP4232	*MATα leu2-3 leu2-112 ura3-52 ino1 gcd6Δ gcn2Δ::hisG Ura3-52::HIS4-lacZ* [*gcd6*(G369V) *CEN LEU2*] [*URA3 CEN*]	Richardson et al., 2004
GP4233	*MATα leu2-3 leu2-112 ura3-52 ino1 gcd6Δ gcn2Δ::hisG Ura3-52::HIS4-lacZ* [*gcd6*(I413A) *CEN LEU2*] [*URA3 CEN*]	Richardson et al., 2004
GP4234	*MATα leu2-3 leu2-112 ura3-52 ino1 gcd6Δ gcn2Δ::hisG Ura3-52::HIS4-lacZ* [*gcd6*(W618R) *CEN LEU2*] [*URA3 CEN*]	Richardson et al., 2004
GP4235	*MATα leu2-3 leu2-112 ura3-52 ino1 gcd6Δ gcn2Δ::hisG Ura3-52::HIS4-lacZ* [*GCD6 CEN LEU2*] [*URA3 CEN*]	Richardson et al., 2004
GP4248	*MATα leu2-3 leu2-112 ura3-52 ino1 gcd6Δ gcn2Δ::hisG Ura3-52::HIS4-lacZ* [*GCD6 CEN LEU2*] [*GCN2 URA3 CEN*]	Richardson et al., 2004
GP4763	*MATα leu2-3 leu2-112 ura3-52 ino1 gcd6Δ gcn2Δ::hisG Ura3-52::HIS4-lacZ* [*gcd6*(D138A) *CEN LEU2*]	Richardson et al., 2004
GP4865	*MATα leu2-3 leu2-112 ura3-52 ino1 gcd6Δ gcn2Δ::hisG Ura3-52::HIS4-lacZ* [*gcd6*(R39A) *CEN LEU2*]	Reid et al., 2012
GP4867	*MATα leu2-3 leu2-112 ura3-52 ino1 gcd6Δ gcn2Δ::hisG Ura3-52::HIS4-lacZ* [*GCD6 CEN LEU2*]	Reid et al., 2012
GP5122	*MAT***a** *leu2-3 leu2-112 trp1-Δ63 ura3-52 GAL2+ gcd1::LEU2 Ura3-52::HIS4-lacZ gcn2Δ* pAV1418 [*GCD1*-FLAG2-His6 *TRP CEN*]	GP lab collection
GP5949	*MATα prb1-1122 pep4-3 leu2 trp1 ura3-52 gal2* pAV1353 [*GCN3 GCD2 GCD7 URA3* 2μm] pAV1413 [*GCD1*-FLAG2-His6 *GCD6 LEU2* 2μm]	GP lab collection
GP7038	*MAT***a** *leu2-3 leu2-112 trp1-Δ63 ura3-52 GAL2+ gcd1::LEU2 Ura3-52::HIS4-lacZ gcn2Δ* [*GCD1 URA3 CEN*] pAV7024 [*gcd1(*K66A)-FLAG2-His6 *TRP1 CEN*]	In this study
GP7039	*MAT***a** *leu2-3 leu2-112 trp1-Δ63 ura3-52 GAL2+ gcd1::LEU2 Ura3-52::HIS4-lacZ gcn2Δ* [*GCD1 URA3 CEN*] pAV7025 [*gcd1(*K66D)-FLAG2-His6 *TRP1 CEN*]	In this study
GP7040	*MAT***a** *leu2-3 leu2-112 trp1-Δ63 ura3-52 GAL2+ gcd1::LEU2 Ura3-52::HIS4-lacZ gcn2Δ* [*GCD1 URA3 CEN*] pAV7026 [*gcd1(*K66R)-FLAG2-His6 *TRP1 CEN*]	In this study
GP7041	*MAT***a** *leu2-3 leu2-112 trp1-Δ63 ura3-52 GAL2+ gcd1::LEU2 Ura3-52::HIS4-lacZ gcn2Δ* [*GCD1 URA3 CEN*] pAV7028 [*gcd1(*D173A)-FLAG2-His6 *TRP1 CEN*]	In this study
GP7042	*MAT***a** *leu2-3 leu2-112 trp1-Δ63 ura3-52 GAL2+ gcd1::LEU2 Ura3-52::HIS4-lacZ gcn2Δ* [*GCD1 URA3 CEN*] pAV7029 [*gcd1(*D173K)-FLAG2-His6 *TRP1 CEN*]	In this study
GP7043	*MAT***a** *leu2-3 leu2-112 trp1-Δ63 ura3-52 GAL2+ gcd1::LEU2 Ura3-52::HIS4-lacZ gcn2Δ* [*GCD1 URA3 CEN*] pAV7030 [*gcd1(*D173N)-FLAG2-His6 *TRP1 CEN*]	In this study

GP7044	*MAT***a** *leu2-3 leu2-112 trp1-Δ63 ura3-52 GAL2+ gcd1::LEU2 Ura3-52::HIS4-lacZ gcn2Δ* pAV7024 [*gcd1(*K66A)-FLAG2-His6 *TRP CEN*]	In this study
GP7045	*MAT***a** leu2-3 leu2-112 trp1-Δ63 ura3-52 GAL2+ *gcd1::LEU2 Ura3-52::HIS4-lacZ gcn2Δ* pAV7025 [*gcd1(*K66D)-FLAG2-His6 *TRP1 CEN*]	In this study
GP7046	*MAT***a** leu2-3 leu2-112 trp1-Δ63 ura3-52 GAL2+ *gcd1::LEU2 Ura3-52::HIS4-lacZ gcn2Δ* pAV7026 [*gcd1(*K66R)-FLAG2-His6 *TRP1 CEN*]	In this study
GP7047	*MAT***a** leu2-3 leu2-112 trp1-Δ63 ura3-52 GAL2+ *gcd1::LEU2 Ura3-52::HIS4-lacZ gcn2Δ* pAV7028 [*gcd1(*D173A)-FLAG2-His6 *TRP1 CEN*]	In this study
GP7048	*MAT***a** *leu2-3 leu2-112 trp1-Δ63 ura3-52 GAL2+ gcd1::LEU2 Ura3-52::HIS4-lacZ gcn2Δ* pAV7030 [*gcd1(*D173N)-FLAG2-His6 *TRP1 CEN*]	In this study
GP7289	*MAT***a** *leu2-3 leu2-112 trp1-Δ63 ura3-52 GAL2+ gcd1::LEU2 Ura3-52::HIS4-lacZ gcn2Δ* pAV1418 [*GCD1*-FLAG2-His6 *TRP CEN*] [*URA3 CEN*]	In this study
GP7290	*MAT***a** *leu2-3 leu2-112 trp1-Δ63 ura3-52 GAL2+ gcd1::LEU2 Ura3-52::HIS4-lacZ gcn2Δ* pAV1418 [*GCD1*-FLAG2-His6 *TRP CEN*] [*GCN2 URA3 CEN*]	In this study
GP7296	*MAT***a** *leu2-3 leu2-112 trp1-Δ63 ura3-52 GAL2+ gcd1::LEU2 Ura3-52::HIS4-lacZ gcn2Δ* pAV2425 [*gcd1(*K66R)-FLAG2-His6 *TRP CEN*] [*GCN2 URA3 CEN*]	In this study
GP7298	*MAT***a** *leu2-3 leu2-112 trp1-Δ63 ura3-52 GAL2+ gcd1::LEU2 Ura3-52::HIS4-lacZ gcn2Δ* pAV2426 [*gcd1(*D173A)-FLAG2-His6 *TRP CEN*] [*GCN2 URA3 CEN*]	In this study
GP7050	*MAT*α *prb1-1122 pep4-3 leu2 trp1 ura3-52 gal2* pAV7033 [*gcd1(*K66D)-FLAG2-His6 *GCD6 LEU2 2µm*] pAV1353 [*GCN3 GCD2 GCD7 URA3* 2µm]	In this study
GP7051	*MAT***α** *prb1-1122 pep4-3 leu2 trp1 ura3-52 gal* pAV7034 [*gcd1(*K66R)-FLAG2-His6 *GCD6 LEU2 2µm*] pAV1353 [*GCN3 GCD2 GCD7 URA3* 2µm]	In this study
GP7321	*MAT***a** *leu2-3 leu2-112 trp1-Δ63 ura3-52 GAL2+ gcd1::LEU2 Ura3-52::HIS4-lacZ gcn2Δ* pAV2371 [*gcd1(*G12V)-FLAG2-His6 *TRP CEN*]	In this study
GP7322	*MAT***a** *leu2-3 leu2-112 trp1-Δ63 ura3-52 GAL2+ gcd1::LEU2 Ura3-52::HIS4-lacZ gcn2Δ* pAV2371 [*gcd1(*L480Q)-FLAG2-His6 *TRP CEN*]	In this study
GP7329	*MAT*α *leu2-3 leu2-112 ura3-52 ino1 gcd6Δ gcn2Δ::hisG Ura3-52::HIS4-lacZ* [*gcd6*(R39D) *LEU2 CEN*]	In this study
GP7330	*MAT*α *leu2-3 leu2-112 ura3-52 ino1 gcd6Δ gcn2Δ::hisG Ura3-52::HIS4-lacZ* [*gcd6*(R39E) *LEU2 CEN*]	In this study
GP7331	*MAT*α *leu2-3 leu2-112 ura3-52 ino1 gcd6Δ gcn2Δ::hisG Ura3-52::HIS4-lacZ* [*gcd6*(R39K) *LEU2 CEN*]	In this study
GP7332	*MAT*α *leu2-3 leu2-112 ura3-52 ino1 gcd6Δ gcn2Δ::hisG Ura3-52::HIS4-lacZ* [*gcd6*(D138K) *LEU2 CEN*]	In this study
GP7333	*MAT*α *leu2-3 leu2-112 ura3-52 ino1 gcd6Δ gcn2Δ::hisG Ura3-52::HIS4-lacZ* [*gcd6*(D138N) *LEU2 CEN*]	In this study
GP7335	*MAT***a** *leu2-3 leu2-112 trp1-Δ63 ura3-52 GAL2+ gcd1::LEU2 Ura3-52::HIS4-lacZ gcn2Δ* pAV2371 [*gcd1(*L480Q)-FLAG2-His6 *TRP CEN*] [URA3 CEN]	In this study

Table S2. Plasmids used.
Related to STAR Methods.

Plasmids	Genotype	Reference
pAV1193 (pRS314)	*TRP1 CEN*	Sikorski and Hieter, 1989
pAV1195 (pRS316)	*URA3 CEN*	Sikorski and Hieter, 1989
pAV1163/ p256	*GCD1 URA3 CEN*	Hill and Struhl, 1988
pAV1198/ p722	*GCN2 URA3 CEN*	Wek et al., 1990
pAV1265/pJB102	*GCD6 LEU2 CEN*	Bushman et al., 1993
pAV1353 (pY24)	*GCN3 GCD2 GCD7 URA3 2µm*	Yang and Hinnebusch, 1996
pAV1413 (pTK11.1)	*GCD1*-FLAG2-His6 *GCD6 LEU2 2µm*	Krishnamoorthy et al., 2001
pAV1418	*GCD1*-FLAG2-His6 *TRP CEN*	Mohammad-Qureshi et al., 2007b
pAV1427	P_{GAL1}-FLAG-His6-*GCD6 URA3 2 µm*	Mohammad-Qureshi et al., 2007a
pAV1739	*gcd6*(V57G) *LEU2 CEN*	Richardson et al., 2004
pAV1740	*gcd6*(I90F) *LEU2 CEN*	Richardson et al., 2004
pAV1741	*gcd6*(R323P) *LEU2 CEN*	Richardson et al., 2004
pAV1742	*gcd6*(G369V) *LEU2 CEN*	Richardson et al., 2004
pAV1743	*gcd6*(I413A) *LEU2 CEN*	Richardson et al., 2004
pAV1744	*gcd6*(R284H) *LEU2 CEN*	Richardson et al., 2004
pAV1745	*gcd6*(W618R) *LEU2 CEN*	Richardson et al., 2004
pAV1857	*gcd6*(D138A) *LEU2 CEN*	Reid et al., 2012
pAV1895	*gcd6*(R39A) *LEU2 CEN*	Reid et al., 2012
pAV2371	*gcd1*(G12V)-FLAG2-His6 *TRP1 CEN*	In this study
pAV2372	*gcd1*(L480Q)-FLAG2-His6 *TRP1 CEN*	In this study
pAV2416	*GCD1 GCD6*-FLAG2 *LEU2 2µm*	Adomavicius et al., 2019
pAV2423	*gcd1*(K66A)-FLAG2-His6 *TRP1 CEN*	In this study
pAV2424	*gcd1*(K66D)-FLAG2-His6 *TRP1 CEN*	In this study
pAV2425	*gcd1*(K66R)-FLAG2-His6 *TRP1 CEN*	In this study
pAV2426	*gcd1*(D173A)-FLAG2-His6 *TRP1 CEN*	In this study
pAV2427	*gcd1*(D173K) -FLAG2-His6 *TRP1 CEN*	In this study
pAV2428	*gcd1*(D173N)-FLAG2-His6 *TRP1 CEN*	In this study
pAV2429	*gcd1*(K66A)-FLAG2-His6 *GCD6 LEU2 2µm*	In this study
pAV2430	*gcd1*(K66D)-FLAG2-His6 *GCD6 LEU2 2µm*	In this study
pAV2431	*gcd1*(K66R)-FLAG2-His6 *GCD6 LEU2 2µm*	In this study
pAV2539	*gcd6*(R39D) *LEU2 CEN*	In this study
pAV2540	*gcd6*(R39E) *LEU2 CEN*	In this study
pAV2541	*gcd6*(R39K) *LEU2 CEN*	In this study
pAV2542	*gcd6*(D138K) *LEU2 CEN*	In this study
pAV2543	*gcd6*(D138N) *LEU2 CEN*	In this study

Figure S1. Competition between purine nucleotides for binding to eIF2B.
Related to Figure 1
(A) Gel of Flag purified proteins used in Figure 1.
(B) Excess GDP can displace ^{32P}GTP from eIF2B.

Figure S2. Control GEF assays.
Related to Figure 2
(A) Example single concentration GEF assay ± eIF2B.
(B) Unlabelled nucleotides (1 mM) do not alter the eIF2B-independent rate of Bodipy-GDP release from eIF2. Mean normalised GDP release rate ± s.e.m (n=3). None are significantly different (T-test) from reactions without nucleotide (– nt)

Figure S3. Alignment and structural models of eIF2B and HNPs.
Related to Figure 3
(A) Structural overview and nucleotide-binding pocket detail of ADP-glucose pyrophosphorylase bound to ATP (pink) (Pdb file 1YP3) showing key conserved nucleotide-binding residues (red surface).
(B) As panel (A) except GDP-mannose pyrophosphorylase bound to GTP (2X60, salmon color).
(C) Clustal W (Sievers et al., 2011) muli-sequence alignment highlighting conservation of nucleotide binding residues in HNPs and eIF2B γ and ε sequences.
(D) Structural overlays of eIF2Bγ (green) with the HNPs shown in panel (A).
(E and F) GTP modelling from 2X60 onto eIF2Bγ (E) and eIF2Bε (F). Structural analysis and images used UCSFChimera software version 1.15 (Pettersen et al., 2004).

Figure S4. Expression and eIF2B complex formation for eIF2Bg K66 and D173 mutants.
Related to Figure 3.
(A) Example western blot of Flag-eIF2Bg from extracts of cells expressing indicated WT or mutant variants as sole source of *GCD1*, except for lethal D173K (marked *), where pre-shuffle cells containing both untagged *GCD1* and Flag-*gcd1-D173K* were analysed. Right, quantification of three replicate experiments relative to a loading control antibody, normalised so WT=1. Error shown is standard deviation from the mean.
(B) Input and Co-IP of eIF2 with flag-eIF2B showing all four untagged eIF2B subunits and eIF2 co-purify with Flag-eIF2Bg in low salt (100 mM KCl) buffer. '–' indicates a WT *GCD1* strain negative control lacking the Flag tag.

Figure S5. No effect of guanine or adenine on growth of other eIF2Bγ or eIF2Bε mutations. Related to Figure 4.
(A) *GCD1*(eIF2Bγ) mutations spotted over a range of nucleotide concentrations ±3AT. 0 and 300 μM concentrations are also shown in Figure 4.
(B) *GCD6* (eIF2Bε) mutations.

Data S1. Source uncropped Western blot images
Relates to Figure 5A
Uncropped blots with anti-Flag and and anti-Sui2 antibodies.

Figure S1

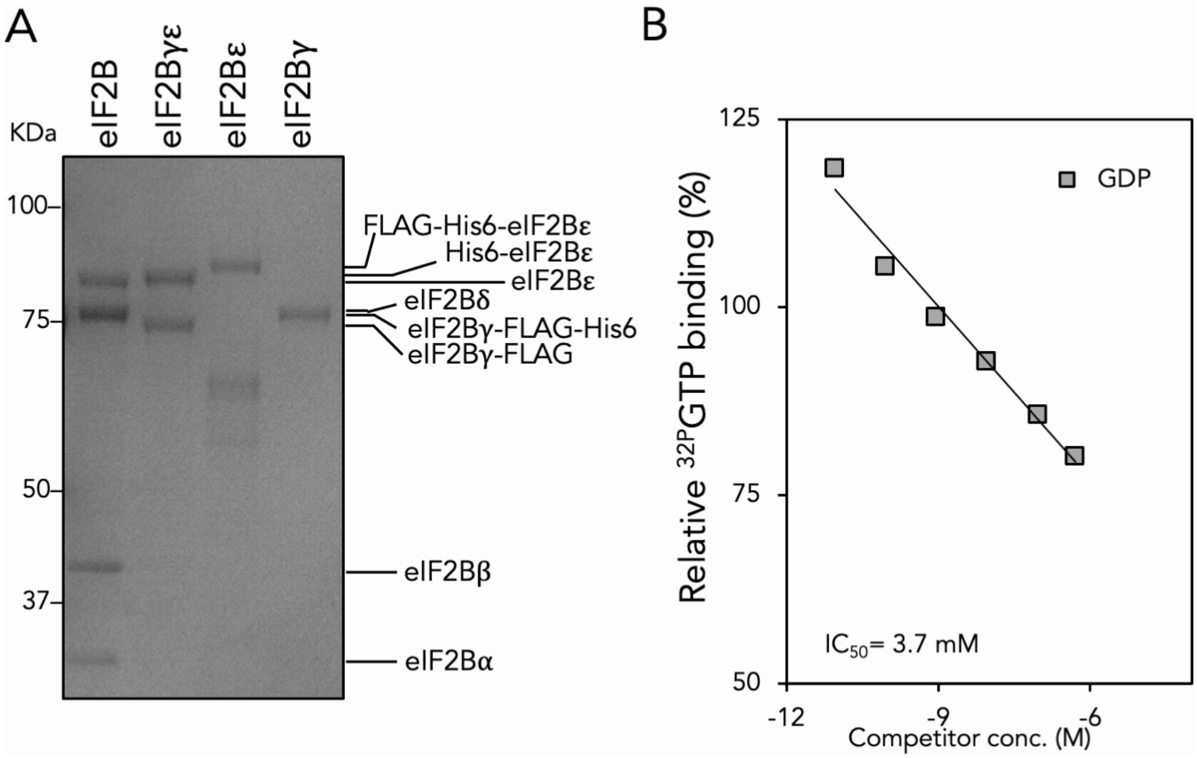
A
B
eIF2B
eIF2Bγε
eIF2Bε
eIF2Bγ
KDa
100
75
50
37
FLAG-His6-eIF2Bε
His6-eIF2Bε
eIF2Bε
eIF2Bδ
eIF2Bγ-FLAG-His6
eIF2Bγ-FLAG
eIF2Bβ
eIF2Bα
125
100
75
50
Relative 32P GTP binding (%)
GDP
IC50= 3.7 mM
-12
-9
-6
Competitor conc. (M)

Figure S2

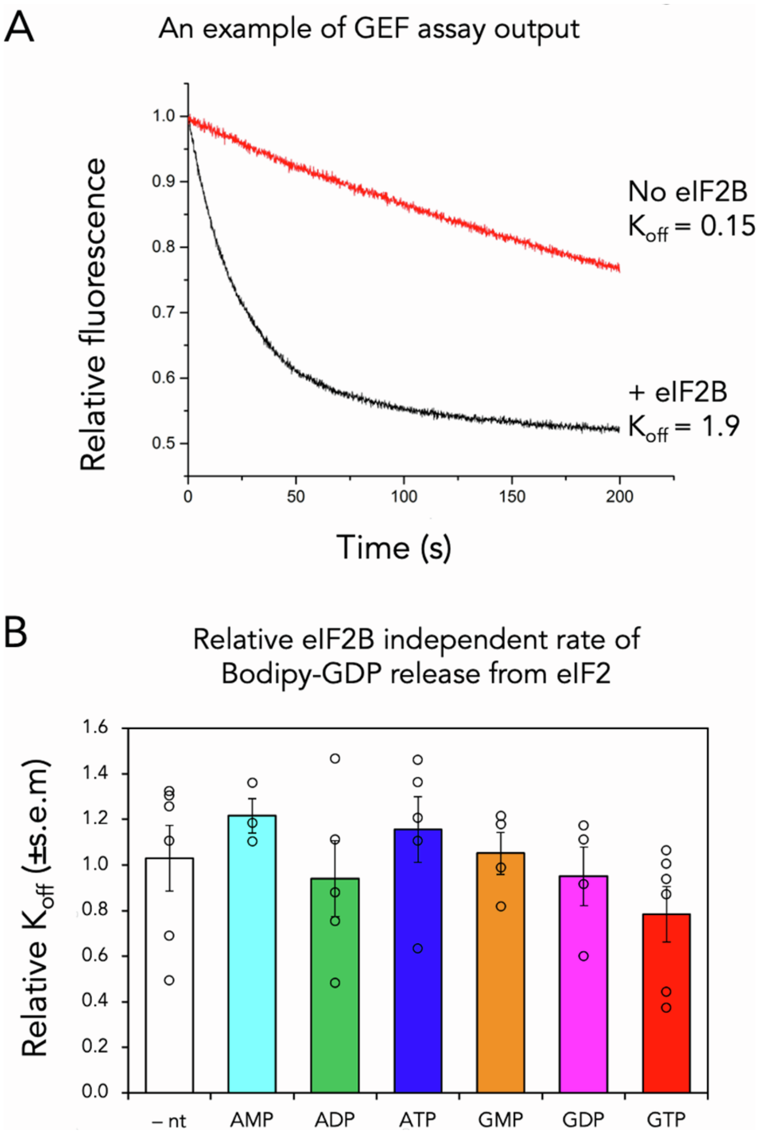
A
An example of GEF assay output
Relative fluorescence
1.0
0.9
0.8
0.7
0.6
0.5
0
50
100
150
200
Time (s)
No eIF2B
K_{off} = 0.15
+ eIF2B
K_{off} = 1.9
B
Relative eIF2B independent rate of
Bodipy-GDP release from eIF2
Relative K_{off} (±s.e.m)
1.6
1.4
1.2
1.0
0.8
0.6
0.4
0.2
0.0
– nt
AMP
ADP
ATP
GMP
GDP
GTP

Figure S3

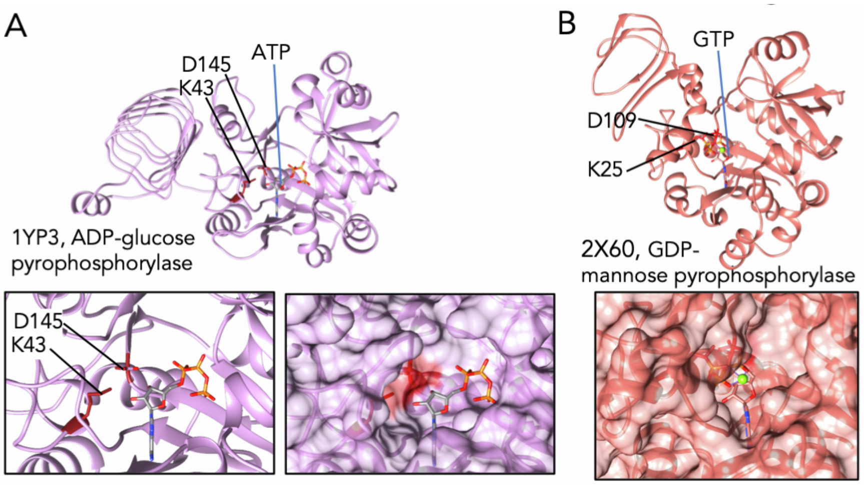

C

```
PPPases                            t tsss.HHHseess            .tTsEEEEEEETTee.t.hhHH
1YP3_AGP_Potato_ATP                L-TKKRAKPAVPLGA 50   (84)  HTVLEYLILAGDHLYRMDYEK 154
2X60_T.mar_GDP-mann-ppase_GTP      LSTPETPKQFLKLFG 32   (65)  DDDEPVLVLPADHRIPDTKKF 118

eIF2Bγ                             .s.sss.hhh.ss s            t..sEEEEEEtteee.s.hhh
2Bγ_S.cerevisiae                   QHSTRLPKALLPI-G 72   (89)  RINGDFVILPCDFVTDIPPQV 182
2Bγ_Sch.pombe                      TGSDALPKALLPI-G 68   (69)  LIKNDFVCLSCDSIVGLPPIY 158
2Bγ_D.melanogast                   V-LGDAPKCLLPV-G 32   (67)  KIKSDFLVVSCDLVSNVSLYP 120
2Bγ_D.rerio                        L-TYNTPKPLLPV-G 31   (68)  KIKTDILVLSCDLITDVALHE 120
2Bγ_X.tropicalis                   L-TASIPKPLLPV-G 33   (67)  KIKTDVLVTSCDLITEVALHE 121
2Bγ_G.gallus                       L-TSSIPKPLLPV-G 31   (62)  KIKTDVLVLSCDLITDVDLYK 114
2Bγ_H.sapiens                      L-TSSIPKPLLPV-G 31   (64)  KLKTDVLVLSCDLITDVALHE 116

eIF2Bε                             t tsss.HHHsbs s            ...s.EEEEEtteee...hhh
2Bε_S.cerevisiae                   L-TAVKPRCLLPL-A 55   (71)  IITGDFILVSGDVLTNIDFSK 147
2Bε_Sch.pombe                      L-TLDKPRCLLPL-A 46   (71)  LITSDFILVSGDVVSNVPLNE 138
2Bε_D.melanogaster                 L-SDEGSTALLPL-V 37   (73)  LIRGHFILLGADTVTNADLRP 131
2Bε_D.rerio                        I-TKDQPRALLPL-A 53   (71)  LVRSDFLLVYGDVVSNVDVSQ 145
2Bε_X.tropicalis                   I-TKDRPRALIPL-A 67   (71)  LVRSDFILVTGDVVSNINIEA 159
2Bε_G.gallus                       I-SKDRPRALLPM-A 57   (71)  LVRSDFILVTGDVVSNLNISR 149
2Bε_H.sapiens                      I-SKDQPRVLLPL-A 71   (71)  LVRSDFLLVYGDVISNINITR 163
```

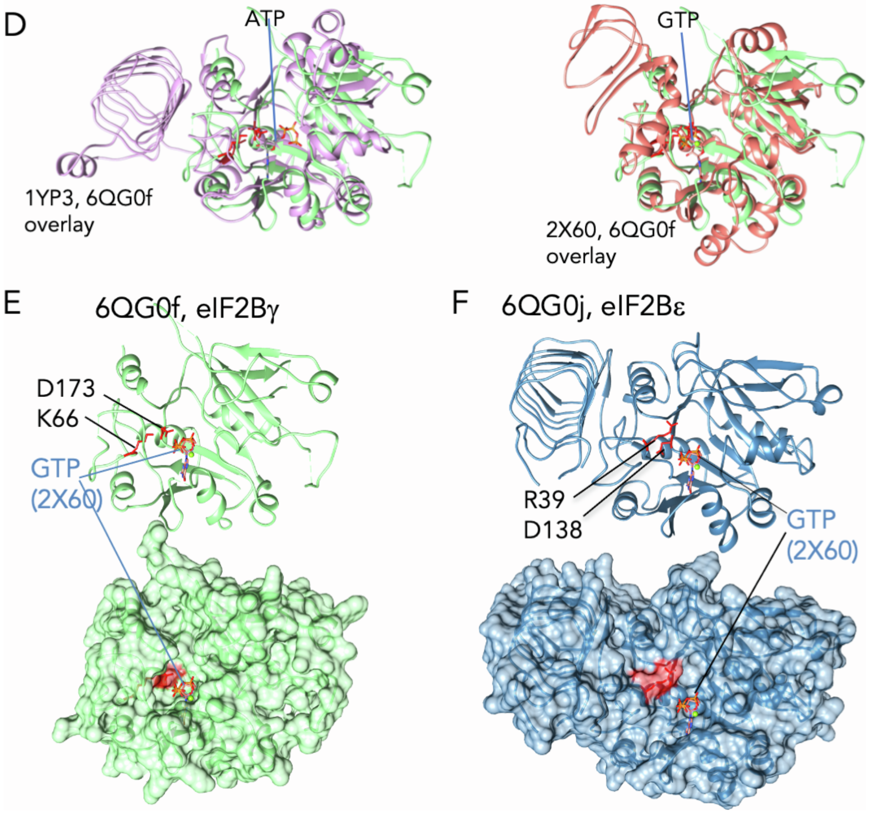

Figure S4

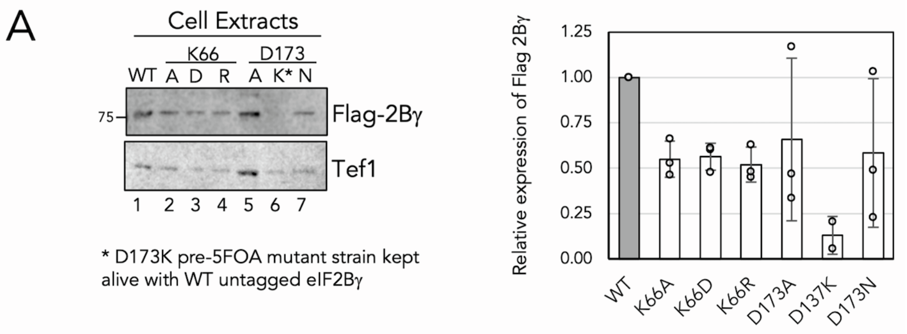
A
Cell Extracts
K66
D173
WT A D R A K* N
75
Flag-2Bγ
Tef1
1 2 3 4 5 6 7
* D173K pre-5FOA mutant strain kept alive with WT untagged eIF2Bγ
Relative expression of Flag 2Bγ
1.25
1.00
0.75
0.50
0.25
0.00
WT
K66A
K66D
K66R
D173A
D137K
D173N

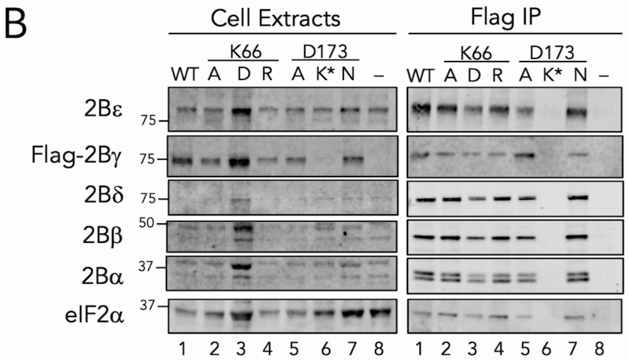
B
Cell Extracts
Flag IP
K66
D173
WT A D R A K* N –
2Bε
Flag-2Bγ
2Bδ
2Bβ
2Bα
eIF2α
75
50
37
1 2 3 4 5 6 7 8

Figure S5

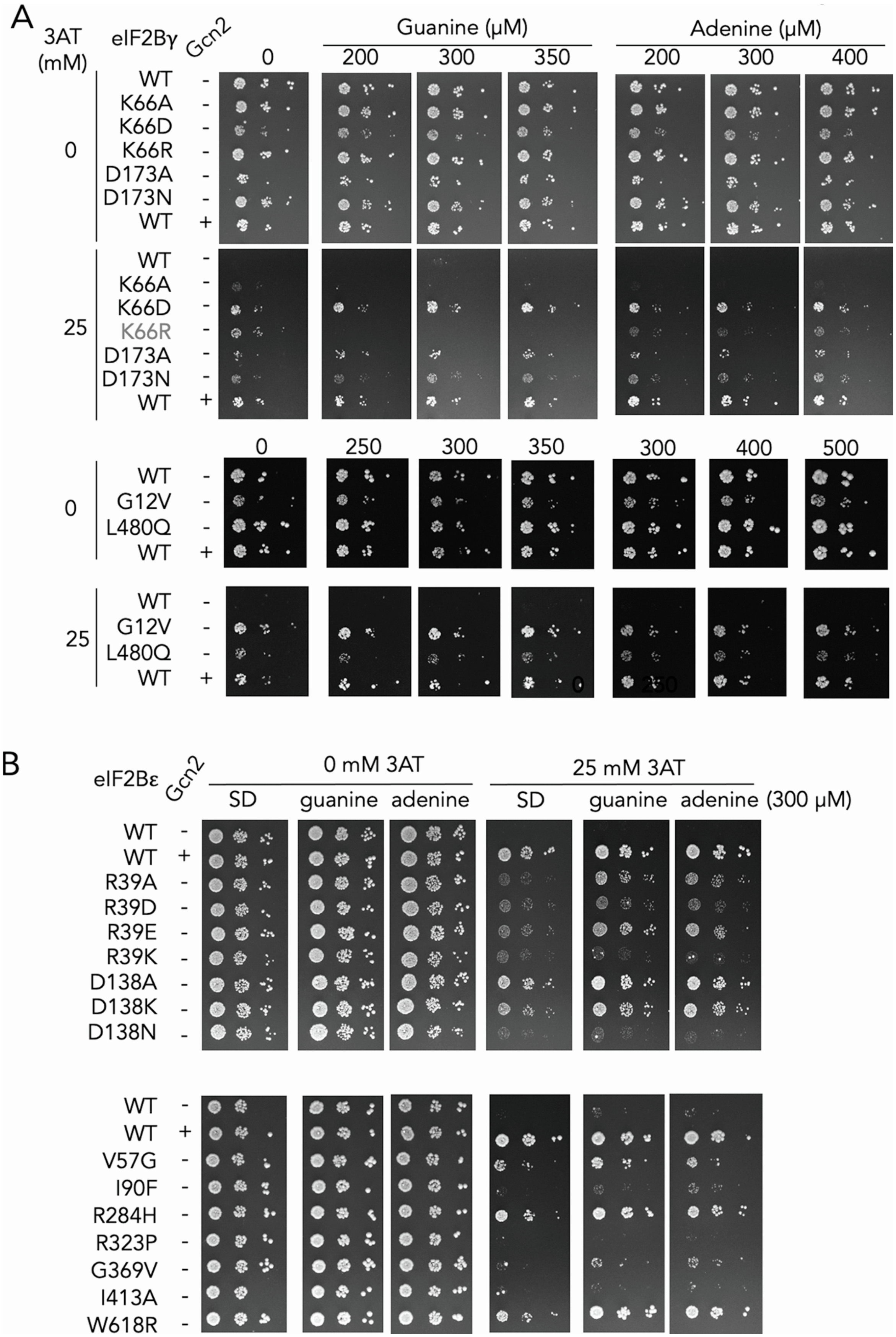
A
3AT (mM)
eIF2Bγ
Gcn2
Guanine (μM)
Adenine (μM)
0
200
300
350
200
300
400
WT -
K66A -
K66D -
K66R -
D173A -
D173N -
WT +
0
25
250
500
G12V -
L480Q -
B
eIF2Bε
Gcn2
0 mM 3AT
25 mM 3AT
SD
guanine
adenine
(300 μM)
WT -
WT +
R39A -
R39D -
R39E -
R39K -
D138A -
D138K -
D138N -
V57G -
I90F -
R284H -
R323P -
G369V -
I413A -
W618R -

Data S1

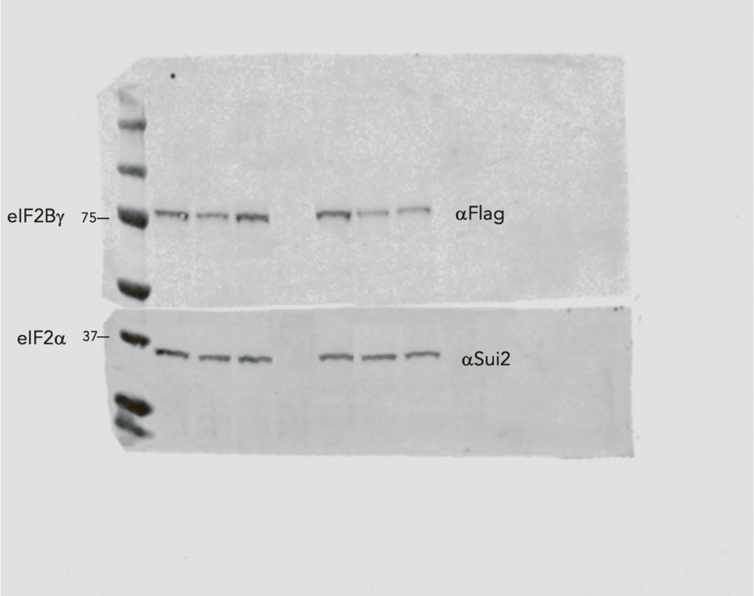

Supplement: Document S1. Figures S1–S5, Tables S1 and S2, and Data S1 [file mmc1.pdf]
